# Use of transcriptomic data for extending a model of the AppA/PpsR system in *Rhodobacter sphaeroides*

**DOI:** 10.1186/s12918-017-0489-y

**Published:** 2017-12-28

**Authors:** Rakesh Pandey, Judith P. Armitage, George H. Wadhams

**Affiliations:** 1 Department of Biochemistry, University of Oxford, South Parks Road, Oxford, UK; 2 Present Address: National Institute of Immunology, Aruna Asaf Ali Marg, New Delhi, India

**Keywords:** Micro-array data, Signal transduction system, Purple non-sulfur bacteria, Photosynthetic bacteria, Mathematical modelling, Gene regulatory network, Oxygen and light sensing

## Abstract

**Background:**

Photosynthetic (PS) gene expression in *Rhodobacter sphaeroides* is regulated in response to changes in light and redox conditions mainly by PrrB/A, FnrL and AppA/PpsR systems. The PrrB/A and FnrL systems activate the expression of them under anaerobic conditions while the AppA/PpsR system represses them under aerobic conditions. Recently, two mathematical models have been developed for the AppA/PpsR system and demonstrated how the interaction between AppA and PpsR could lead to a phenotype in which PS genes are repressed under semi-aerobic conditions. These models have also predicted that the transition from aerobic to anaerobic growth mode could occur via a bistable regime. However, they lack experimentally quantifiable inputs and outputs. Here, we extend one of them to include such quantities and combine all relevant micro-array data publically available for a PS gene of this bacterium and use that to parameterise the model. In addition, we hypothesise that the AppA/PpsR system alone might account for the observed trend of PS gene expression under semi-aerobic conditions.

**Results:**

Our extended model of the AppA/PpsR system includes the biological input of atmospheric oxygen concentration and an output of photosynthetic gene expression. Following our hypothesis that the AppA/PpsR system alone is sufficient to describe the overall trend of PS gene expression we parameterise the model and suggest that the rate of AppA reduction in vivo should be faster than its oxidation. Also, we show that despite both the reduced and oxidised forms of PpsR binding to the PS gene promoters in vitro*,* binding of the oxidised form as a repressor alone is sufficient to reproduce the observed PS gene expression pattern. Finally, the combination of model parameters which fit the biological data well are broadly consistent with those which were previously determined to be required for the system to show (i) the repression of PS genes under semi-aerobic conditions, and (ii) bistability.

**Conclusion:**

We found that despite at least three pathways being involved in the regulation of photosynthetic genes, the AppA/PpsR system alone is capable of accounting for the observed trends in photosynthetic gene expression seen at different oxygen levels.

**Electronic supplementary material:**

The online version of this article (10.1186/s12918-017-0489-y) contains supplementary material, which is available to authorized users.

## Background

Induction and repression of PS genes that encode proteins required for the formation of photosynthesis apparatus in the purple non sulfur bacterium *Rhodobacter sphaeroides* depends on redox and light signals. These PS genes mainly consist of the *puc* (encoding structural and assembly proteins of light harvesting complex II), *puf* and *puh* (encoding structural and assembly proteins of light harvesting complex I and reaction centre), *bchl* (encoding enzymes involved in bacteriochlorophyll biosynthesis) and *crt* (encoding enzymes involved in carotenoid biosynthesis) operons [1, 2]. In the presence of sufficient oxygen (≈ 200 μM dissolved oxygen concentration), *R. sphaeroides* uses respiration to generate energy and PS gene expression is almost completely repressed. When oxygen is reduced below a certain level (≤ 3 μM dissolved oxygen concentration) they switch to photosynthetic growth and the extent of PS gene expression depends on the available light intensity [3]. In addition, *R. sphaeroides* can undergo anaerobic respiration using a terminal electron acceptor such as DMSO (dimethyl sulfoxide) [4].

**Fig. 1:**
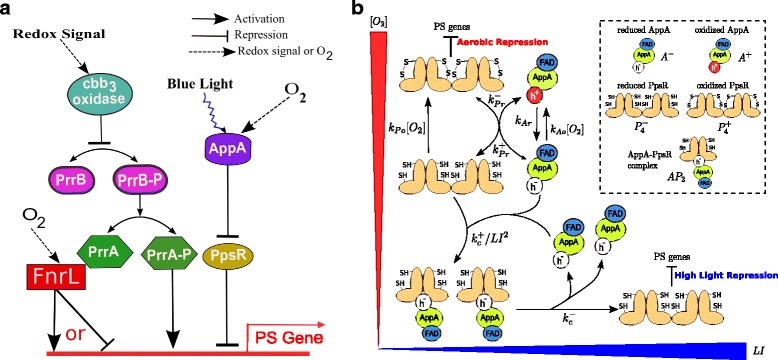
Schematic diagrams for photosynthetic gene regulation and the existing model for the AppA/PpsR system. **a** A diagram of light and redox dependent regulation of photosynthesis genes by PrrB/A, AppA/PpsR and FnrL in *Rhodobacter sphaeroides*. Note that the FnrL system activates PS genes expression under anaerobic condition, in cooperation with PrrB/A, while it inhibits expression under aerobic conditions. Here, the red thick line with red arrow on top denotes expression, black arrows represent activation, and the line with bar end denotes inhibition of PS genes. The bidirectional arrow represents the phosphorylation and dephosphorylation of PrrB and PrrA. A dotted and zigzag line shows sensing of oxygen / redox and blue light, respectively. **b** The simple model for the light and redox dependent interaction of AppA and PpsR (copied from Pandey et al., 2011 [26] with permission)**.** Here, [O_2_] represents the oxygen concentration and LI blue light irradiance. Oxidised and reduced forms of PpsR are denoted by tetrameters with disulfide bonds (S-S) and thiol groups (S-H), respectively. AppA has two cofactors attached: (i) FAD, and (ii) heme. The h^+^ and h^−^ represent oxidised and reduced form of heme cofactor, respectively. The lines with bar end denote repression of PS genes by PpsR, and the thickness of that shows the strength of repression; oxidised form of PpsR (PpsR_ox_) is a strong repressor compared to the reduced form (PpsR_red_)

In *R. sphaeroides,* PS gene expression is under the control of three main transcriptional regulatory systems: PrrB/A (RegB/RegA) [5, 6], AppA/PpsR [7] and FnrL [8, 9]. A schematic diagram illustrating the mechanism of their regulation is shown in Fig. 1a. PrrB/A is a two component global regulatory system which activates the expression of PS genes under anaerobic conditions. The FnrL system also induces PS gene expression under anaerobic conditions while the AppA/PpsR system is a repressor of PS gene expression under aerobic conditions. Recently two new regulators of photosynthesis gene expression, CrpK and MppG (RSP_2888) have been suggested by Imam et al., [10, 11] but the mechanisms of regulation by these transcription factors are not yet known. They have also experimentally demonstrated an overlapping nature of the CrpK and FnrL regulons [10].

The two-component system, PrrB/A consists of a histidine sensor kinase PrrB and its cognate response regulator, PrrA. Under anaerobic conditions, PrrB autophosphorylates and transfers the phosphoryl group to PrrA [12]. Phosphorylated PrrA binds the promoter of PS genes and activates their expression. Under aerobic conditions, electron flow through the *cbb3-oxidase* inhibits the kinase activity of PrrB and enhances its phosphatase activity [13], thus preventing activation of PS gene expression by PrrA. It has also been suggested that photosynthetic electron transport during anaerobic growth reduces the electron flow through *cbb3-oxidase* that results in activation of PS genes under these conditions [14].

The AppA/PpsR system is composed of two proteins, PpsR and AppA. At high oxygen levels PpsR binds the upstream region of PS genes and inhibits the expression of these genes [2, 15, 16]. It has been proposed that oxygen directly increases the binding affinity of PpsR by forming an intramolecular disulfide bond in PpsR [17]. Under anaerobic conditions this bond is reduced which results in a lower binding affinity of PpsR for PS gene promoters, thereby relieving repression and allowing PS gene expression. It has been shown that the disulfide bond reduction of PpsR is mediated by a flavoprotein, AppA [17]. However, the exact mechanism underlying this observation is still elusive and unsettled. For example, it has been shown that the C-terminus of AppA is capable of reducing the disulfide bonds in PpsR [18], but on the other hand deletion of this domain does not abolish the antirepressor properties of AppA [19, 20]. According to Masuda and Bauer, AppA regulates the activity of PpsR in two ways; (i) by reduction of the oxidised form of PpsR as described above and (ii) by reduced AppA forming a complex with reduced PpsR (AppA-PpsR_2_) [17]. They have also shown that this complex formation is inhibited by blue-light illumination [17]. Initially it was assumed that this complex is transcriptionally inactive but a recent study suggests that it could bind DNA and form a ternary complex AppA-PpsR_2_-DNA [20]. AppA is capable of sensing both oxygen and blue light using heme [19, 21] and flavin adenine dinucleotide (FAD) cofactors, respectively [22–24]. It is proposed that the light and redox dependent interaction of AppA and PpsR is responsible for a phenotype in which PS gene expression is repressed by blue light under semi-aerobic conditions [17, 23, 25–27].

The FnrL system is composed of a single protein, FnrL, which is a homologue of FnR (a global anaerobic regulator) of *Escherichia coli* [8, 9, 28]. An *fnrL* deletion mutant strain is unable to grow photosynthetically or anaerobically using DMSO as a terminal electron acceptor in the dark [9, 29]. FnrL exerts its regulatory effect by binding the FnrL consensus sequence TTGTCN_4_TTCAA [8]. Under aerobic conditions, FnrL represses expression of the *hemA* gene (encodes 5-aminolevulinate synthase) and *puc* operons while under anaerobic condition it activates expression of both operons [28]. It has been proposed that the FnrL control of PS gene expression is additive and co-operative with the PrrB/A system [28].

Recently, two mathematical models for AppA/PpsR regulation of PS gene expression in *R. sphaeroides* have been developed [26, 27]. These models suggest that the phenotype where PS genes are repressed by blue light at intermediate oxygen levels will occur if PpsR is reduced on a faster time scale than AppA. In both models, authors demonstrated that in the steady state response curve of reduced PpsR a maximum developed at intermediate oxygen levels and it was proposed that this maximum is responsible for the specific repression of PS genes under semi-aerobic conditions.

The first and simple model, shown in Fig. 1b, predicted that in addition to the above mentioned condition, if (i) the copy number of AppA is higher than that of PpsR by at least a factor of two, (ii) the electron transfer from AppA to PpsR is effectively irreversible and (iii) the rate of reoxidation of PpsR is faster than that of AppA then the transition from aerobic to anaerobic growth conditions would occur via a bistable regime [26]. This model was mainly based on the experimental observations that: (i) reduced AppA can reduce oxidised PpsR, and (ii) under low oxygen levels reduced PpsR and reduced AppA form a transcriptionally inactive complex and that this complex formation is inhibited by blue light [26].

A subsequent study extended the first model [26] by incorporating a more detailed description of blue light regulation [27]. In addition to the prediction of the development of a maximum (role explained above) and bistability, the model results were compared with available data for PS gene expression inhibition at varying blue light irradiance under semi-aerobic conditions. Also the extended model was able to explain the reduced blue light sensitivity of an *appA* mutant.

Crucially, it is important to note that both models excluded the roles of PrrB/A and FnrL in PS gene regulation. In addition, the models have an input (K_O_), which compares the rate of oxidation and reduction of AppA, and output (PpsR _red/ox_) neither of which are measurable in vivo*.* Features such as bistability also require specific values for certain parameters in the model such as the rate of reduction of PpsR being faster than that of AppA, and the copy number of AppA being greater than that of PpsR by at least a factor of two [26]. Many parameters, including the rate of reduction and oxidation of AppA and PpsR, are not experimentally tractable in vivo. The major limitation of these models is that due to above reasons we cannot directly compare and test the model predictions although micro-array data are publically available for PS gene expression under diverse growth condition. Therefore, we need a model that has measurable inputs (oxygen level and or light intensity) and outputs (e.g. PS gene expression level).

Here, we set out to extend one of these models and incorporate the biologically relevant input (oxygen concentration) and output (relative level of a PS gene, *pucB,* transcription), which will allow us to estimate the unknown model parameters using micro-array data. We combined all relevant micro-array data sets available for a PS gene (*pucB*) of this bacterium.

## Results

### Combining the publically available micro-array data sets for *pucB* gene

**Fig. 2:**
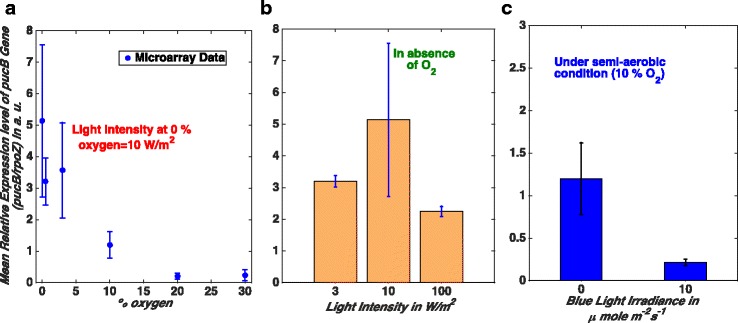
Transcriptomic patterns of *pucB* gene obtained from combining publically available micro-array data. Here, gene expression data is shown with the symmetric error of one standard deviation. **a** We present the data obtained after normalisation and combination of the micro-array data. The filled circles represent the mean relative expression of *pucB* at different oxygen levels. In absence of oxygen, cells are grown in white light of 10 W/m^2^ light intensity and under semi-aerobic and aerobic conditions cells are grown in the dark. **b** The inhibition of *pucB* expression by high intensity white light in absence of oxygen. The data presented here are obtained from our analysis of the micro-array data. **c** The data obtained from our analysis that shows the known repression of PS genes by blue light illumination at an intermediate oxygen level (10% oxygen). Note that, like panel (**a**) the data presented in panels (**b**) and (**c**) come from multiple studies

We normalised and combined the various publically available micro-array datasets for a PS gene expression under different growth conditions. We collected MAS 5.0 (Microarray Suite 5.0) values provided for the gene expression levels in NCBI (National Center for Biotechnology Information) Gene Expression Omnibus (GEO) database which are already normalised values for a submitted data series. The MAS 5.0 is a name of the algorithm used for producing gene expression level by Affymetrix. However, in order to compare the data from different data series, often from different research groups, we had to re-normalise that. We chose *pucB* (locus tag RSP 0314 and probe id 1194–1198) of the *puc* (or *pucBA*) operon as a representative PS gene as it is highly represented with five copies in the probe set of the *R. sphaeroides* 2.4.1 genechip [30]. It encodes polypeptides of the B800–850 light harvesting complex (LH II) [31]. We used *rpoZ*, encoding the DNA-directed RNA polymerase ω subunit to normalise the gene expression levels of *pucB* across diverse genechip datasets. *rpoZ* was chosen because: (i) the expression level of *rpoZ* (locus tag RSP 1669, probe id 2587) from the same genechip data series remains almost unchanged under anaerobic, semi-aerobic and aerobic growth regimes (Additional file 1: Figure S1), and (ii) *rpoZ* has previously been used as a control in qPCR reactions validating the genechip data [30]. We collected and analysed all micro-array gene expression data for *pucB* and *rpoZ* submitted to the GEO database by different research groups for *R. sphaeroides* 2.4.1 under diverse growth conditions. We omitted the data submitted by Arai et al., [32] as their data for 2% oxygen level shows a high standard deviation. The obtained mean relative expression levels and the obtained gene expression pattern are shown in Fig. 2a. The obtained transcriptomic profile supports the general hypothesis that under anaerobic conditions in the presence of low light (≈ 10 W/m^2^) *pucB* is highly expressed to facilitate photosynthesis, whereas it is strongly repressed under aerobic conditions. However, in the presence of high intensity white light (≈ 100 W/m^2^) under anaerobic conditions, *pucB* expression is reduced compared to that at 10 W/m^2^ (Fig. 2b), which is consistent with the previous study [3]. A similar pattern has also been reported for gene RSP3361 (encoding putative restriction endonuclease or methylase) which is positively regulated by PrrA [33]. In addition, Fig. 2c confirms the phenotype in which *pucB* is repressed by blue light illumination under semi-aerobic conditions [34]. Overall, our methodology for normalising and combining diverse micro-array datasets appears to recapitulate the known and observed biological phenotypes validating this approach.

### Extending the existing model to have biologically relevant inputs and outputs

**Fig. 3:**
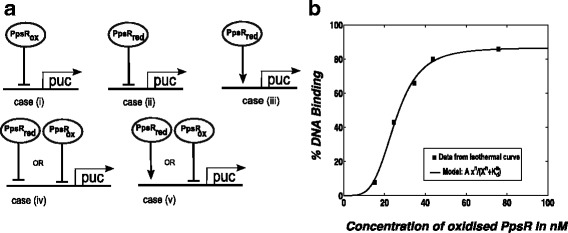
Schematics for the PpsR-DNA binding possibilities and estimation of the Hill coefficient for the oxidised PpsR. **a** The arrow and line with bar end represent activation and repression, respectively. The solid thick line with arrow on top denotes the expression of PS genes. Abbreviations: red: reduced form, ox: oxidised form. **b** Estimating the Hill coefficient for the binding of oxidised PpsR to a DNA fragment containing the *puc* promoter. Here, we fit the data obtained from binding isotherms for oxidised PpsR published elsewhere [17]. We use a general Hill function of the form Ax^n^/(K_d_^n^ + x^n^) to fit the data

In order to compare the existing model [26, 27] to experimental data (obtained from our data analysis) we had to extend the simple model [26] to include the biologically relevant input (atmospheric oxygen concentration) and output (relative levels of photosynthetic gene transcription). The output from the previous model (summarized in  Additional file 1 and published elsewhere [26]) is concentration of reduced PpsR (PpsR_red_) or oxidized PpsR (PpsR_ox_), which is not quantifiable in vivo. To extend our model to have the physiologically relevant output of *pucB* expression it is necessary to model the interaction of PpsR with the *puc* promoter. PpsR is thought to regulate photosynthetic gene expression by binding to two consensus palindromic PpsR binding sites, TGTcN_10_gACA (N is nucleotide and lower case letters represent lesser conservation) upstream of these genes [2, 15, 16, 35]. There are several possibilities for the role of the binding of PpsR molecules to these binding sites. It has been observed that in vitro, both forms of PpsR (PpsR_ox_ and PpsR_red_) can bind a DNA fragment containing the tandem PpsR binding site with different binding affinities [17]. The in vitro EC_50_ (50% DNA binding) for PpsR_ox_ and PpsR _red_ are 31 nM and 69 nM, respectively [17]. In addition, it has been reported that PpsR represses PS gene expression, but we do not know which form of PpsR (oxidised or reduced) binds in vivo or whether one or both forms bind to achieve gene regulation. We therefore modelled the binding of PpsR_ox_ and PpsR_red_ to the *pucB* promoter and used Hill functions to model these protein-DNA interactions. As our purpose was to allow us to estimate the main parameters for the established model we did not consider more complex cases, for example those in which AppA-PpsR_2_ could also compete with various forms of PpsR for DNA binding. We therefore investigated five initial possibilities for the PpsR-*puc* binding (Fig. 3a) of the general form:

$$ Level of pucB mRNA={M}_{max}\left(\kern0.28em \frac{{\mathrm{K}}^{\mathrm{n}}\kern0.28em }{\kern0.28em {\mathrm{K}}^{\mathrm{n}}+{\mathrm{PpsR}}^{\mathrm{n}}\kern0.24em }\right)\kern5.40em (1) $$

where M_max_ is maximum expression level of *pucB* gene, n is the Hill coefficient and K is the EC_50_ value for binding between PpsR and the *puc* promoter. The EC_50_ for the binding of PpsR_ox_ has been estimated 31 nM and the binding of PpsR_red_ as 69 nM through in vitro experiments [17]. From the obtained transcriptomic data shown in Fig. 2a, we suggest the maximal *pucB* expression level under anaerobic conditions to be 5.1 in arbitrary unit (a.u.). Therefore, we believe that the 5.1 a. u. could be a reasonable estimate of M_max_. For PpsR_ox_ we estimated a Hill coefficient (n_1_) = 4.1 from curve fitting of the data for the isothermal curve published elsewhere [17] (Fig. 3b). For PpsR_red_ the value for the Hill coefficient (n_2_) = 3.4 was estimated in a previous study [27]. A full description of all five models and parameters are given in  Additional file 1.

Note that for all following results obtained using this extended model, we assume that the electron transfer from AppA to PpsR is effectively irreversible (i.e. parameter K_eq_ = ∞, see Additional file 1). Also, we do not attempt to estimate the parameter δ (defined in the  Additional file 1 and elsewhere [26]) of the model as it has been shown that the steady state behaviour of the AppA/PpsR system is independent of it [26].

**Fig. 4:**
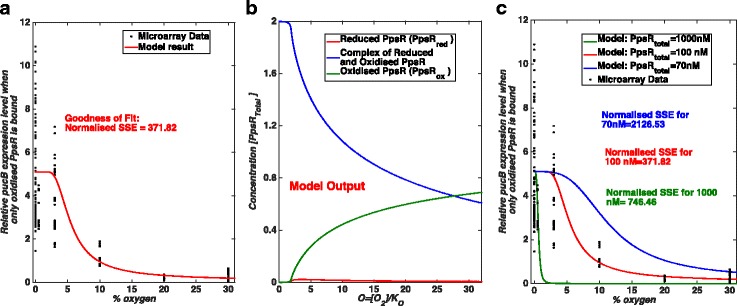
Case (i) when only PpsR_ox_ binds and acts as a repressor of PS gene expression. Here, we show normalised SSE (sum of squared error) values as a measure of the goodness of fit. The normalised SSE values are calculated as described in the Method section. The black filled circles show the micro-array data obtained from our analysis. **a** Comparison of our model results with the obtained experimental data. The model best recapitulates the trends of *pucB* gene expression for parameters: *I* = 0.001, α = 6, β = 500, γ = 2.1, δ = 1, and K_eq_ = ∞ (see parameter definitions in Additional file 1 and Table 1). Here, we have assumed that 30% O_2_ ≅ 200–380 μM and 0% O_2_ = 0 μM as well as the total concentration of PpsR (PpsR_total_ )= 100 nM. **b** A model output for the parameter combination used in panel **a**. It is to show that for the above combination of model parameters, the free (i.e. not bound to AppA) concentration of the reduced form of PpsR is very low in fact negligible. **c** The shape of the curve for different total PpsR concentrations (PpsR_total_). Note that in panel (**a**) and (**c**) level of oxygen is shown in terms of % of oxygen in the gas mixture bubbled in the culture during experiments. In panel (**b**) oxygen level is represented by a dimensional parameter O as it shows the simulation result obtained from the existing simple model [26]

**Fig. 5:**
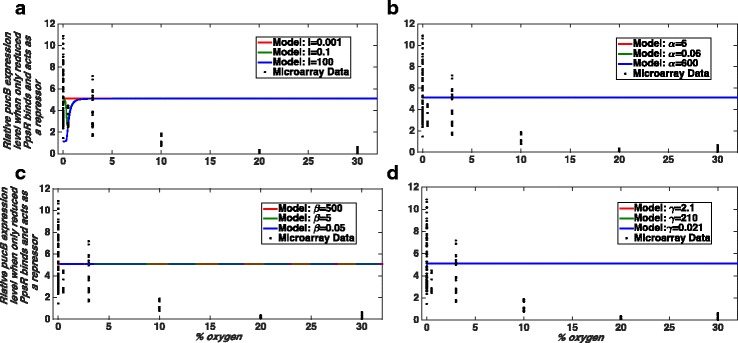
The *pucB* gene expression pattern when PpsR_red_ binds the *puc* promoter and acts as a repressor of that (Case (ii)). Here, we compare the *pucB* gene expression pattern obtained from our modelling and micro-array data analysis. The black filled circles show the experimental data obtained from our micro-array data analysis which we compare with model results for varying parameters one by one. **a** Effect of changes in blue light irradiation (*I)* on the shape of the curve obtained from the simulation. **b** Effect of changes in parameter α on the shape of the curve obtained from the simulation. Similarly (**c**) and (**d**) present changes in the *pucB* gene expression pattern if we change the parameter values of β and γ, respectively. Note that the model result for the parameter combination which would fit the data very well is shown by red coloured solid lines in all panels. That particular parameter combination is considered as the default parameter values in all simulations. The default parameters are:* I *= 0.001, α = 6, β = 500, γ = 2.1, δ = 1, and K_eq_ = ∞. Like in Fig. 4, we have assumed that 30% O_2_ ≅ 200–380 μM and 0% O_2_ = 0 μM as well as the total concentration of PpsR is assumed to be 100 nM. We have increased and/or decreased each parameter value by at least two orders of magnitudes from their default values. The model results in this case are unable to explain the experimental gene expression pattern. Here, we do not feel the need of calculating normalised SSE as from the visual inspection we do not see any hope for a good data fitting

**Fig. 6:**
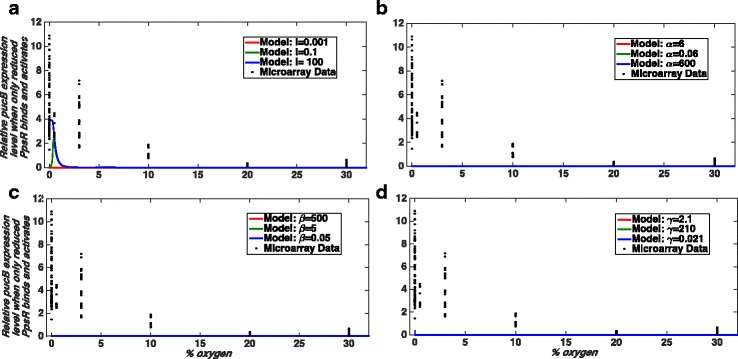
Case (iii) when PpsR_red_ binds the *puc* promoter and acts as an activator of that. Similar to the Fig. 5 here we show how our model results vary if the value of a particular parameter is increased and/or decreased from their default values and those are compared with the experimental data. The black filled circles show the micro-array data. Model results for varying parameter values are shown in different panels as: *I *in **a**, α in **b**, β in **c **and γ in **d**. We see that like case (ii) the model results in this case also unable to explain the experimental gene expression pattern. Here, similar to Fig. 5 we do not calculate normalised SSE for each curve as from visual inspection we do not see any hope for good data fitting. The default parameters are: *I*= 0.001, α = 6, β = 500, γ = 2.1, δ = 1, and K_eq_ = ∞. Here, we have assumed that 30% O_2_ ≅ 200–380 μM and 0% O_2_ = 0 μM as well as the total concentration of PpsR is assumed to be 100 nM

Our model results suggest that the binding of PpsR_ox_ as a repressor (case i) or in combination with PpsR_red_ binding as a co-repressor (case iv) or as a co-activator (case v) would be capable of reproducing the experimentally observed pattern of *pucB* gene expression under certain parameter combinations (Fig. 4a, Additional file 1: Figure S2 and S3). However, the binding of PpsR_red_ alone to the promoter as either a repressor (case ii) or activator (case iii) of expression (*puc*) would not be compatible with the experimentally observed pattern of *puc* gene expression (Figs. 5 and 6). Given that PpsR_red_ has been shown to bind in vitro to the promoter with an affinity which is only half of that of PpsR_ox_ [17], we set out to investigate why it appeared to have so little effect on *puc* expression within our models. We determined the levels of free PpsR_red_, free PpsR_ox_ and the amount of PpsR which is complexed with AppA in our model at different oxygen concentrations for case (i); the outcome is shown in (Fig. 4b). Based on that we suggest that the reason that PpsR_red_ binding has so little effect on *puc* expression in this case is due to the very low levels of free PpsR_red_ in a cell under these conditions which are defined by the combination of model parameters that fit the data. It should be noted that we do not propose that PpsR_red_ can be ignored, only suggest that in wild-type cells most PpsR_red_ is complexed with AppA under these conditions (Fig. 4b). This result is entirely consistent with the observation that an AppA null mutant was unable to grow under photosynthetic conditions [36] as in the absence of AppA, all of the PpsR_red_ will be free and hence able to bind and repress transcription of the PS genes. For this reason, we continued our studies using the simplest model in which PpsR_ox_ alone binding to the *puc* promoter represses its transcription.

### Estimating biological parameters of the established model

We started from the hypothesis that the AppA/PpsR system alone might account for the observed pattern of photosynthesis gene expression under semiaerobic conditions. This supposes that the PrrB/A and FnrL systems are involved in setting the absolute levels of expression but do not influence the shape of the transition curve at intermediate oxygen concentrations (or over all shape of the gene expression pattern).

In the existing simple model for AppA/PpsR regulation ([26], summarized in Additional file 1), oxygen concentration is expressed in units of the parameter K_O_ which is defined as

$$ {K}_o=\frac{kAr}{kAo}=\frac{rate constant for the reduction of AppA}{rate constant for the oxidation of AppA} $$

The unit of the rate constant for the oxidation of App (k_Ao_) is: sec^−1^/unit of O_2_ and unit of the rate constant for the rate of reduction of AppA (k_Ar_) is sec^−1^. This leads to the conclusion that the unit of the parameter K_O_ is the unit of oxygen level in the present study.

The experimental range of oxygen level is from 0 to 30% of the mixture of the gas that is bubbled into the culture while generating the micro-array data. In the experiments in order to mimic the aerobic condition researchers have used a mixture of gas that contains 30% oxygen, 69% N_2_, and 1% CO_2_, for low oxygen condition a mixture of 3% O_2_, 96%N_2_, and 1% CO_2_ and for anaerobic condition a mixture of 99% N_2_, and 1% CO_2_. However, experimentalists have used another definition of the aerobic, semi-aerobic and anaerobic conditions in terms of dissolved oxygen in the medium and there is a general understanding that ≈ 200 μM dissolved oxygen constitutes aerobic, ≈ 100 μM semi-aerobic and <3 μM anaerobic conditions [14, 23, 26].

In order to calculate a value range for Ko we have assumed that the oxygen levels in the aerobic samples are between the experimentally assumed concentration of ~ 200 μM and the saturation limit of oxygen in water at 30 °C which is ~ 380 μM. Previously, when cultures were grown in 60 mL glass tubes and sparged with a gas mixture containing 10% O_2,_ 89% N_2_ and 1% CO_2_, researchers have claimed that it is equivalent to 120 μM dissolve oxygen concentration [25]. This means that the maximum oxygen level, 30% oxygen in the experiment, is equivalent to the maximum value of the parameter O of the model, which is a dimension less quantity. In our model the fully aerobic condition is defined as being when the concentration of free PpsR_ox_ accounts for more than 50% of the PpsR_total_ and the PS genes are fully repressed for the parameter combination which fits the data well. From Fig. 4 this can be seen to be when O ≅ 30. Therefore, this maximum value of the parameter O will be between 200 μM/K_O_ and 380 μM/K_O_ resulting in a value range for the parameter K_O_ ≈ 6.7–12.7 μM. Because the value of K_O_ > 1, we would suggest that the rate of reduction of AppA is faster than its rate of oxidation.

Note that the output from our model is designed to be insensitive to the exact value of K_O_ or the oxygen concentration in the aerobic culture as these are both related and are absorbed into the dimensionless parameter O (see Eq.2 in  Additional file 1 or Eq. 9 in [26]). Thus, whether the concentration of dissolved oxygen in the cultures representing our aerobic conditions are 200 μM or 380 μM will only change the value of K_O_ and not the parameter O. This is because O = oxygen concentration/K_O_ and so if the assumed oxygen concentration under aerobic conditions is slightly larger, K_O_ will also be larger to compensate. Therefore, increasing the assumed concentration of dissolved oxygen only serves to give a larger value for Ko and hence more likelihood that the rate of reduction of AppA is faster than the rate of its oxidation.

**Fig. 7:**
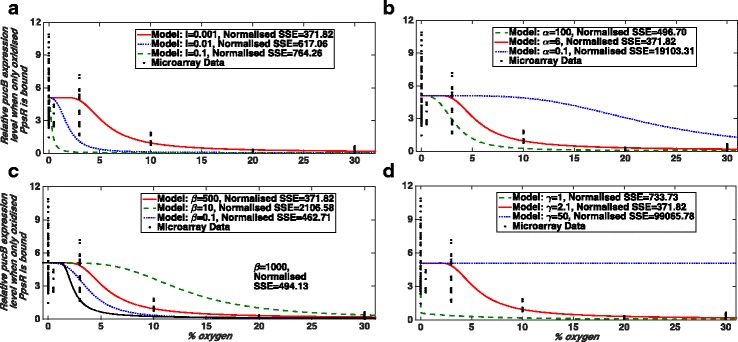
Effects of changes in model parameters one by one for the Case (i). Similar to Figs 5 and 6 model results for varying parameter values are shown in different panels and compared with the experimental data. Here, like in previous figures, black filled circles show the micro-array data. **a** Effect of changes in blue light irradiance (*I)*. **b** Simulation results for different values of parameter α. **c** Effect of changes in β. **d **Model outputs for varying values of γ. We consider following model parameter combination as a default combination*: I* = 0.001, α = 6, β = 500, γ = 2.1, δ = 1, and K_eq_ = ∞. Again, we have assumed that 30% O_2_ ≅ 200–380 μM and 0% O_2_ = 0 μM as well as the total concentration of PpsR is assumed to be 100 nM. A solid red line in each panel shows the model output for the default parameter combination. The normalised SSE here, are calculated as described in the Method section and are used to quantify the goodness of the fit

Using our full model from atmospheric oxygen concentration as an input to *puc* gene expression as an output we evaluated the effects of changing each parameter in the model in turn. Biologically, the parameter *I* represents light irradiance of blue light. The parameter α compares the rate of re-oxidation of PpsR with that of AppA. The parameter β represents the time scale separation in the rate of reduction of PpsR and that of AppA. The parameter γ represents the ratio of the copy number of AppA and PpsR in a single cell. The steady state behaviour of the model is independent of the parameter δ (published elsewhere [26] and defined in  Additional file 1) [26]. Figure 7 shows the results of varying each parameter in our full model for case (i). We find that the model best recapitulates the observed biological data when *I* is very small (around 0.001), α is around 6, β is around 500 and γ is 2.1. We also found that the total concentration of PpsR (PpsR_total_), which is a parameter in the new model, should be approximately 100 nM (Fig. 4c). The recommended parameter combination is summarised in Table 1.

**Table 1:** The main model parameters and their recommended values based on this study

Model Parameters	Biological Meaning	Recommended Values
α	Compares the rate of re-oxidation of PpsR with that of AppA	6
β	Compares the rate of reduction of PpsR with that of AppA	500
γ	Ratio of the concentration of AppA and PpsR in a single cell	2.1
PpsR_total_	Total concentration of PpsR	≈100 nM
K_O_	Compares the rate of reduction of AppA with its rate of oxidation	≈ 6.7–12.7 μM
*I*	Blue Light Irradiance	0.001

**Fig. 8:**
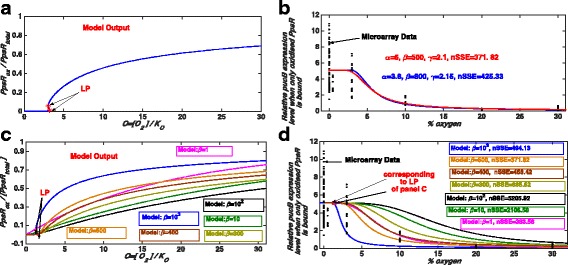
Bistability, another model parameter combination and model outputs for different values of β. As in previous figures, black filled circles show the micro-array data. **a** and **b** Another parameter combination for which the model result is consistent with the micro-array data along with the prediction of a bistable behaviour. Here, LP denotes a limit point bifurcation. Model parameters for **a** are: *I* = 0.001, α = 3.8, β = 800, γ=2.15, δ=1, and K_eq_ = ∞. Parameters for **b** are: *I* = 0.001, δ=1, K_eq_ = ∞ and PpsR_total_ = 100 nM. Again here we have assumed that 30% O_2_ ≅ 200–380 μM and 0% O_2_ = 0 μM. Normalised SSE values are calculated as described in the Method section. **c** and **d** Appearance of bistability and changes in the gene expression profile obtained from the full model for different values of β. Here, LP and nSSE denote a limit point bifurcation and normalised SSE, respectively. Model parameters are: *I* = 0.001, α = 6, γ = 2.1, δ = 1, K_eq_ = ∞ and PpsR_total_ = 100 nM. Here also, we have assumed that 30% O_2_ ≅ 200–380 μM and 0% O_2_ = 0 μM. Limit points (LP) are for β = 1000 only

In order to determine which parameter combination leads to a better fit and recapitulate the observed data, we calculated normalised SSE (sum of squared error) values for each curve obtained from changing the model parameters. This value indicates how good our model predictions are (the method is explained in the Method section) and a smaller value of it suggests a better fit. We find that for this parameter combination we have a relatively small normalised SSE value. However, there could be other model parameter combinations for which model results are consistent with the experimental data (although with a larger normalised SSE) for example for *I* = 0.001, α = 3.8, β = 800 and γ=2.15 (Fig. 8a and b).

An *I* value of 0.001 is consistent with the fact that the biological data were from cells grown either in the dark or under 10 W/m^2^ white light and hence there should only be a small component of blue light present to elicit a blue light response. An α value >1 (e.g. 6 or 3.8) suggests that the rate of re-oxidation of PpsR is larger than that of AppA. This was shown to be one of the key requirements for bistability to exist in this system on the transition between aerobic and anaerobic growth conditions in the original model [26]. A γ value of 2.1 suggests that the copy number of AppA in the cell should be roughly twice that of PpsR. This was also shown to be a requirement for the system to show bistability in the original model [26]. A large β value (of 500 or 800) suggests that the rate of reduction of PpsR is faster than that of AppA, in vivo which supports the previous model prediction (published elsewhere [26]). Note that the simple model of AppA/PpsR system predicted that the phenotype in which PS genes are repressed under semi-aerobic conditions can occur if β is large. Therefore, we suggest that such phenotype at intermediate oxygen level would be due to the dominant role of the AppA/PpsR system under those conditions. These results also suggest that the AppA/PpsR system alone might account for the observed trend of PS gene expression under semi-aerobic conditions.

In addition, our α, β and γ values are consistent with those required for the system to show bistability [26]. According to the original model [26], bistability in the aerobic ↔anaerobic growth transition requires a large β. However, examining the effects of increasing β (Figs. 7c, 8c and d) shows an interesting trend. When we increase β up to a value of about 100 we find that the fraction of oxidised PpsR decreases at each given oxygen concentration, and hence provides a progressively worse fit to the experimental data (relatively higher values of normalised SSE). However, increasing β beyond 100 results in the opposite trend in that the fraction of oxidised PpsR starts to increase again for each given oxygen concentration (Fig. 8c), and therefore progressively better fit (Fig. 8d). Whilst our best fit for the higher β value is 500, a value at which bistability is still not observed for the system (Figs. 4b, 7c and 8b), there could be other parameter combinations for which the model results would fit the data equally well. It is therefore not possible to preclude the fact that there could be another combination of parameters which fit the experimental data with β large enough for bistability to exist in this system, for example *I* = 0.001, α = 3.8, β = 800 and γ=2.15 (Fig. 8a and b). Such inability to find a unique parameter value through fitting experimental data is not new, has been extensively studied and is widely known as the parameter identifiability issue [37–40]. In our case, with the current data, the identifiability issue means that we cannot unambiguously estimate a unique value of β. Therefore, based on this study we cannot confirm or reject the possibility of bistability in this system. In fact, we do not aim to make such decisions here; for that more comprehensive studies should be performed. However, we wish to convey that modelling is essential to obtain these parameters as some of them such as α, β and K_O_ are not tractable to experimental measurement.

## Discussion

Regulation of the transition between aerobic and photosynthetic growth requires one of the largest changes in gene expression profiles for bacteria. Despite extensive study by both experimental and mathematical modelling we still don’t have a complete understanding of how this regulation is achieved, even in the well-characterised bacterium *Rhodobacter sphaeroides*. Moreover, there is a vast amount of micro-array data available for this bacterium. This is partly due to the fact that various research groups have measured PS gene expression under the environmental conditions required for their particular study, and therefore there is no complete dataset for PS gene expression under all relevant oxygen and light levels.

Here, we obtained publically available micro-array datasets from diverse research groups and combined them. Note that we used the MAS 5.O value of the gene expression which is a normalised gene expression level itself but we had to re-normalise as we wanted to compare the data sets from different data series (from different research groups). This allowed us to generate a pattern of PS gene expression across a wide range of oxygen and light levels. Our normalised and combined data recapitulates the known biology of the system such as increased repression of PS genes as the concentration of oxygen increases (Fig. 2a), reduced PS gene expression with increased light intensity (Fig. 2b) in the absence of oxygen and decreased PS gene expression under semi-aerobic growth in the presence of blue light (Fig. 2c).

Many areas of biology are benefiting from advances in mathematical modelling. However, there are still significant challenges in parameterising these models using biological data. Here, we present a method to extend mathematical models to include biologically relevant inputs and outputs and then to use normalised microarray data to estimate parameters which cannot be experimentally determined. We applied our procedure to the well-studied pathway regulating bacterial photosynthetic gene expression in *Rhodobacter sphaeroides.* Taking the availability of mathematical models of AppA/PpsR and published transcriptomic data into consideration we extended an existing model of the AppA/PpsR system to include the biologically meaningful input of oxygen concentration and output of relative PS gene expression. In order to estimate the parameters of that established model, we compared the data obtained from our micro-array data analysis with the extended model. We observed that the trend in PS gene expression at different oxygen concentrations could be described by the model for AppA/PpsR regulation alone. This suggests that the AppA/PpsR system could be primarily responsible for regulating gene expression trends in response to changing oxygen conditions under semi-aerobic conditions. Also, the model of AppA/PpsR system would work in biologically relevant range of oxygen (0 to 30%) if the rate of reduction of AppA is faster than its rate of oxidation.

Previous analysis of the existing model for AppA/PpsR regulation of PS gene expression showed that there are a range of parameters for which the system would show bistability [26] and that there are areas of parameter space in which bistability could exist in the system provided certain conditions are met. Our model parameterisation shows that one of the best fits to the experimental data are when the γ ~ 2.1, α ~ 6 and β is ~500. This parameter combination is consistent with the model parameters required in the previous study for the occurrence of PS gene repression at intermediate oxygen levels and bistablity. Hence, the present study supports our previous model prediction that the PS genes can indeed be repressed under semi-aerobic conditions in high blue light illumination if PpsR is reduced on a faster time scale than AppA. Whilst, to the best of our knowledge, bistability has not been observed to date in this system although it is a common phenomenon in biology and has, for example, been observed in sugar uptake systems of *Escherichia coli* [41, 42]. Given the steep nature of oxygen gradients in the environment and the length of time it takes for a bacterium to change from aerobic to photosynthetic growth, bistability could provide a distinct advantage to cells experiencing rapidly fluctuating oxygen levels under semi-aerobic conditions.

Furthermore, our model predicts that the total concentration of PpsR in the cell should be around 100 nM (Fig. 4c) and as our estimated value for γ is approximately 2, the total concentration of AppA should be around 200 nM, a value which could be experimentally validated. It should be noted that the absolute concentration of AppA should be dependent on oxygen levels as *appA* gene is indirectly regulated by the PrrB/A system [43].

The major limitation of this study is that we are not able to confirm or reject the model prediction of bistablity. Furthermore the current microarray data are not sufficient to overcome the identifiability issues we have, especially for the parameter β. In addition, we have shown the gene expression pattern of only one PS gene out of several. However, one can carry out similar studies to get the transcriptomic pattern of other PS genes for diverse environmental conditions.

## Conclusions

We have demonstrated that our method of combining micro-array data for a PS gene is able to recapitulates the known biology of the system. Also, we presented a method to extend mathematical models to include biologically relevant inputs and outputs and then to use normalised micro-array data to estimate parameters which cannot be experimentally determined. We applied our procedure to the well-studied pathways regulating bacterial photosynthetic gene expression in *Rhodobacter sphaeroides*, extending the model to include available oxygen as the biological input and gene expression as the output. We found that despite at least three pathways being involved in this regulation, one regulatory pathway (AppA/PpsR) alone could be able to account for the observed trends in photosynthetic gene expression measured under semi-aerobic growth conditions. Parameters determined from comparing results from an extended model of the system with normalised micro-array data from a range of research groups are broadly consistent with those previously determined to be required for the AppA/PpsR system to show bistability in vivo. In addition, parameterisation of the extended model using the data obtained from our analysis suggests that the rate of reduction of AppA would be faster than its rate of oxidation.

This study demonstrates how combining mathematical modelling with experimental data can provide insights into a systems behaviour which are not tractable by either approach alone. This general framework of model extension could be useful for other systems. This study also highlights the need for further experimental data allowing a new and extended version of the mathematical model to incorporate cross talk between the AppA/PpsR, FnrL and PrrBA systems along with the suggestion of the formation of an AppA-PpsR_2_-DNA ternary complex at the *puc* promoter.

## Methods

### Combining the micro-array data from different data series

We obtained the micro-array data from Gene Expression Omnibus (GEO) database of National Center for Biotechnology Information (NCBI) (http://www.ncbi.nlm.nih.gov/geo/). The gene expression data available there are in raw and normalised both forms. They have used either MAS 5.0 or RMSA (Robust Multi-Array Analysis) method of normalisation. In the present study we have used the MAS 5.O value for the gene expression level from GEO. In order to compare the data from different data series (or for normalisation), we first calculated a relative expression level of *pucB* in each data sample. For this, we divided the *pucB* gene expression level with the *rpoZ* gene expression level for each data sample. After that, these relative *pucB* expression levels from different samples of different data series are compared to get the transcriptomic pattern of *pucB* gene for different oxygen levels and light intensities.

### Calculating the normalised sum of squared error

We calculate a normalised SSE (sum of squared error) value for each transcriptomic pattern obtained from the extended model for a particular combination of parameters to decide how good that model prediction is. In general, a SSE value which is also called SSR (sum of squared residuals) indicates how well a model result is fitting the data. We use following expression to calculate SSE for a particular oxygen level.

$$ \mathrm{SSE}={\sum}_{i=1}^n{\left({y}_i-Y\right)}^2 $$

where n denotes number of data points at a particular oxygen level (% oxygen). A y_*i*_ value denotes a gene expression level for the *i* ^th^ data point at a particular oxygen level in the micro-array data. A model predicted value of the gene expression level at that particular oxygen level is represented by Y. Therefore, the total SSE is

$$ \mathrm{Total}\kern0.24em \mathrm{SSE}={\sum}_{j=1}^6{\sum}_{i=1}^n{\left({y}_i-{Y}_j\right)}^2 $$

Here, j denotes a particular oxygen level. In our data we have total 6 oxygen levels. Note that in our transcriptomic data, number n also varies for each oxygen levels.

We observe that in our experimental gene expression pattern variance at each oxygen level is different. In fact, variance is very high at low oxygen levels, and therefore the normalisation is required so that each data point has a comparable contribution for the total SSE. We normalise the individual SSE for a particular oxygen level by dividing it with variance of the micro-array data at that oxygen level.

$$ \mathrm{Nomalised}\kern0.24em \mathrm{SSE}\kern0.24em \mathrm{for}\kern0.24em {\mathrm{j}}^{\mathrm{th}}\%\mathrm{level}\  \mathrm{of}\  \mathrm{oxygen}=\frac{\sum_i^n{\left({y}_i-{Y}_j\right)}^2}{\sigma_j^2} $$

where *σ* represents standard deviation.

Finally, we calculate normalised SSE for a gene expression pattern obtained from the model using following expression.

$$ \mathrm{Normalised}\kern0.24em \mathrm{SSE}={\sum}_{j=1}^6\left(\mathrm{Nomalised}\;\mathrm{SSE}\kern0.24em \mathrm{for}\kern0.24em {\mathrm{j}}^{\mathrm{th}}\%\mathrm{level}\  \mathrm{of}\  \mathrm{oxygen}\right) $$

or

$$ \mathrm{Normalised}\kern0.24em \mathrm{SSE}={\sum}_{j=1}^6\left(\frac{\;\sum \limits_{i=1}^n{\left({y}_i-{Y}_j\right)}^2}{\sigma_j^2}\right) $$

A normalised SSE value close to zero suggests that the curve obtained from the modelling fits the micro-array data very well. Therefore, a smaller value of it indicates a better fit.

### Softwares and tools

All computational work has been done using MATLAB [44] and MATLAB toolboxes: (i) MATCONT [45] and (ii) Curve Fitting Tool Box.

## Additional files

Additional file 1: Figure S1. Gene expression pattern of *rpoZ* at different oxygen levels and light intensities. We see that the expression of *rpoZ* is independent of environmental conditions. **Figure S2**. Case (iv) when both PpsR_ox_ and PpsR_red_ are able to bind the *puc* promoter and both act as repressor. **Figure S3**. Case (v) when in vivo both oxidised and reduced PpsR are able to bind the promoter region of *pucB* but PpsR_ox_ represses whereas PpsR_red_ activates transcription. (DOCX 1003 kb)

Additional file 2: List of micro-array data series used for this study and data. (DOCX 118 kb)

## Abbreviations

DMSO: Dimethyl Sulfoxide
GEO: Gene Expression Omnibus
MAS 5.0: Microarray Suite 5.0
NCBI: National Center for Biotechnology Information
Ox: Oxidised
PS: Photosynthetic
Red: Reduced
RMSA: Robust Multi-Array Analysis
SSE: Sum of Squared Error
