# Supplementary material for: Use of transcriptomic data for extending a model of the AppA/PpsR system in Rhodobacter sphaeroides
Source: BMC Syst Biol. 2017 Dec 28;11:146. doi: 10.1186/s12918-017-0489-y (PMC5747161; doi:10.1186/s12918-017-0489-y)
Supplement: Supplementary file 1 — Gene expression pattern of rpoZ at different oxygen levels and light intensities. We see that the expression of rpoZ is independent of environmental conditions. Figure S2. Case (iv) when both PpsRox and PpsRred are able to bind the puc promoter and both act as repressor. Figure S3. Case (v) when in vivo both oxidised and reduced PpsR are able to bind the promoter region of pucB but PpsRox represses whereas PpsRred activates transcription. (DOCX 1003 kb) [file 12918_2017_489_MOESM1_ESM.docx]

**Additional file 1**

**Use of transcriptomic data for extending a model of the AppA/PpsR system in *Rhodobacter sphaeroides***

Rakesh Pandey^+*^, Judith P. Armitage, George H. Wadhams ^*^

Department of Biochemistry, University of Oxford, South Parks Road, Oxford, UK

^*^ Corresponding author

George H. Wadhams george.wadhams@bioch.ox.ac.uk

Rakesh Pandey rpandey@nii.ac.in

^+^ Present address is

National Institute of Immunology, Aruna Asaf Ali Marg, New Delhi - 110067, India

**1. The simple model**

In what follows we describe the simple mathematical model for AppA/PpsR in short that is published elsewhere (1). This model was developed based mainly on two experimental observations (i) AppA dependent reduction of oxidised PpsR (P_4_^+^), and (ii) light inhibited complex formation between reduced AppA (A^-^) and reduced PpsR (P_4_^-^). For explaining the model and writing an ordinary differential equation system for that we use following notations

A^-^ = reduced form of AppA , A**^+^** = oxidised form of AppA, P_4_^-^ = reduced form of PpsR, , P_4_^+^ = oxidised form of PpsR, and AP_2 =_ complex of AppA and PpsR

where superscript 4 denotes that the protein exists as a tetramer. This model considers following processes through which AppA and PpsR interact with each other.

**The Reduction of oxidised PpsR by reduced AppA**

The reduced form of AppA (A^-^) reduces a disulfide bond (S-S) in oxidised PpsR (P_4_^+^) and it occurs independently of the light conditions. In order to investigate the change in phenotype if this process is reversible, this process was modelled as a following reversible event although biochemically it is thought to be irreversible (2).

A^-^ *+* P_4_^+^ $\begin{matrix} \underset{\to}{k_{Pr}^{+}} \\ \overset{\leftarrow}{k_{Pr}^{-}} \end{matrix}$ A**^+^** + P_4_^-^

Where A**^+^** represents the oxidised form of AppA. The $k_{Pr}^{+} \mathrm{and}k_{Pr}^{-}$ are second order rate constants and the equilibrium constant ($K_{eq}$) defined as $K_{eq}=\frac{k_{Pr}^{+}}{k_{Pr}^{-}}$.

**The complex formation between PpsR and AppA**

The light dependent complex formation is modelled as

2A^-^ *+* P_4_^-^ $\begin{matrix} \underset{\to}{k_{c}^{+}/{LI}^{2}} \\ \overset{\leftarrow}{k_{c}^{-}} \end{matrix}$ 2AP_2_

where AP_2_ denotes the formed complex. This description takes into account the observed 2:1 stoichiometry and the light-dependent inhibition of the complex formation between PpsR and AppA. Here, $k_{c}^{+}/{LI}^{2}$ and $k_{c}^{-}$represent an effective third-order rate constant and a second-order rate constant, respectively. The rationale behind the inverse quadratic dependence of the forward rate on light irradiance is explained elsewhere (1).

**The oxygen dependent redox regulation of AppA**

Since, the exact molecular mechanism of the redox sensing of AppA is unknown, the redox regulation of AppA was modelled as

A**^+^** $\begin{matrix} \underset{\to}{k_{Ar}} \\ \overset{\leftarrow}{k_{Ao}[O_{2}]} \end{matrix}$ A^-^

Here, it was assumed that in absence of oxygen, the heme cofactor attached to AppA is constitutively reduced by some unknown mechanism with rate constant k_Ar_. It was also assumed that the oxidation of heme occurs proportional to the oxygen concentration with rate constant $k_{Ao}[O_{2}]$ where $[O_{2}]$ denotes the oxygen concentration.

**Reoxidation of PpsR**

It was argued that if the reduction of PpsR by AppA is an irreversible reaction then there should a process through which reduced PpsR is oxidised and that should be independent of AppA. Due to the lack of information about such process it was modelled as

P_4_^-^ $\underset{\to}{k_{Po}{[O]}_{2}}$ P_4_^+^

where it was assumed that the rate of reoxidation of PpsR is proportional to oxygen concentration and the corresponding rate constant is $k_{Po}{[O]}_{2}$.

If we write mass action kinetics for the above mentioned set of biochemical reactions then we get the following ordinary differential equation system (for detail see Pandey et. al (1) )

$$\frac{{d[A}^{-}]}{dt}=k_{Ar}\left[ A^{+} \right]-k_{Ao}\left[ O_{2} \right]\left[ A^{-} \right]-k_{Pr}^{+} \left[ A^{-} \right]\left[ P_{4}^{+} \right]+ k_{Pr}^{-}\left[ A^{+} \right]\left[ P_{4}^{-} \right]-2\left( \frac{k_{c}^{+}}{{LI}^{2}}\left[ A^{-} \right]^{2}\left[ P_{4}^{-} \right]-k_{c}^{-} {{[AP}_{2}]}^{2} \right)$$

$$\frac{{d [P}_{4}^{-}]}{dt}=k_{Pr}^{+} \left[ A^{-} \right]\left[ P_{4}^{+} \right]-k_{Pr}^{-}\left[ A^{+} \right]\left[ P_{4}^{-} \right]-k_{Po}\left[ O_{2} \right] \left[ P_{4}^{-} \right] - \left( \frac{k_{c}^{+}}{{LI}^{2}}\left[ A^{-} \right]^{2}\left[ P_{4}^{-} \right]-k_{c}^{-} {{[AP}_{2}]}^{2} \right) \left( 1 \right)$$

$$\frac{d{[AP}_{2}]}{dt}=2\left( \frac{k_{c}^{+}}{{LI}^{2}}\left[ A^{-} \right]^{2}\left[ P_{4}^{-} \right]-k_{c}^{-} {{[AP}_{2}]}^{2} \right)$$

Here, it was assumed that the total amount of PpsR and AppA protein are conserved according to

$\left[ P_{4}^{+} \right]+\left[ P_{4}^{-} \right]$ +$\frac{1}{2}$ [${AP}_{2}$] =[$P_{T}$] and

$\left[ A^{-} \right]+[A^{+}]$ +[${AP}_{2}$] =[$A_{T}$]

We could change the Equation (1) into a dimensionless ordinary differential equation (ODE) system as following

$$\frac{{dx}_{1}}{d\tau}=1-x_{1}\left( 1+O \right)-\frac{x_{3}}{\gamma}-2\frac{\delta}{\gamma} \left( x_{1}^{2}x_{2}-I^{2}\frac{x_{3}^{2}}{\gamma^{2}} \right)-- \frac{\beta}{\gamma}\left[ x_{1}\left( 1-x_{2}-\frac{x_{3}}{2} \right)-\frac{x_{2}}{K_{eq}}\left( 1-x_{1}-\frac{x_{3}}{\gamma} \right) \right]$$

$\frac{{dx}_{2}}{d\tau}=\beta\left[ x_{1}\left( 1-x_{2}-\frac{x_{3}}{2} \right)-\frac{x_{2}}{K_{eq}}\left( 1-x_{1}-\frac{x_{3}}{\gamma} \right) \right]-\alpha Ox_{2}-\delta(x_{1}^{2}x_{2}-I^{2}\frac{x_{3}^{2}}{\gamma^{2}})$ (2)

$$\frac{{dx}_{1}}{d\tau}=2\delta\left( x_{1}^{2}x_{2}-I^{2}\frac{x_{3}^{2}}{\gamma^{2}} \right)$$

where time ($\tau$) is measured in units of 1/k_Ar_. Also, $x_{1}=\frac{A^{-}}{[A_{T}]}$, $x_{2}=\frac{[P_{4}^{-}]}{[P_{T}]}$, $x_{3}=\frac{[{AP}_{2}]}{[P_{T}]}$, $x_{4}=\frac{[P_{4}^{+}]}{[P_{T}]}$, $x_{5}=\frac{[A^{+}]}{{[A}_{T]}}$ where A_T_ and P_T_ denote total amount of AppA and PpsR, respectively.

Definitions of the parameters of Eq.2 are

$\alpha=\frac{k_{Po}}{k_{Ao}}$ , β =$\frac{k_{Pr}^{+}[A_{T}]}{k_{Ar}}$ , 𝛾 =$\frac{{[A}_{T}]}{{[P}_{T}]}$ , 𝛿 =$\frac{k_{c}^{+}{[A_{T}]}^{2}}{{LI}^{2} k_{Ar}}$ , $K_{eq}=\frac{k_{Pr}^{+}}{k_{Pr}^{-}}$ , O =$\frac{{[O}_{2}]}{K_{o}}$ , I =$\frac{LI}{K_{L}}$ , $K_{O}$ =$\frac{k_{Ar}}{k_{Ao}}$ , $K_{L}={(\frac{k_{c}^{+}P_{T}}{k_{c}^{-}})}^{1/2}$.

**2 Expression Pattern of *rpoZ* (Fig S1)**

**
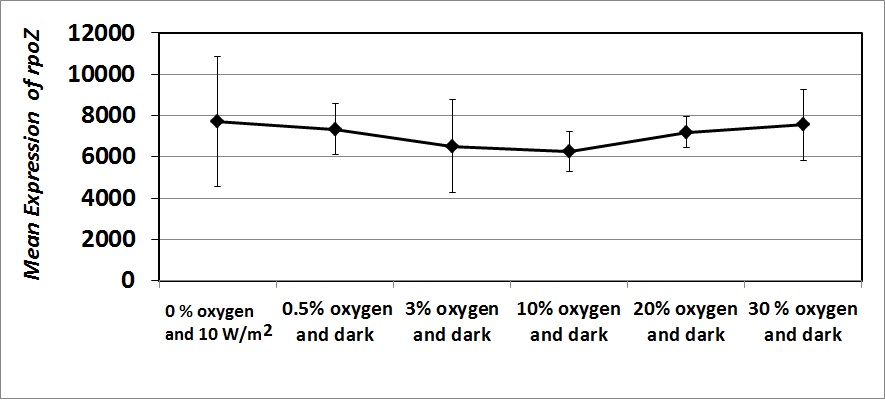
**

**Figure S1**. Gene expression pattern of *rpoZ* at different oxygen levels and light intensities. From this figure it is clear that the expression of *rpoZ* is independent of environmental conditions.

**3 Possible Models for PpsR-*puc* Promoter Binding**

We model the binding of PpsR to the *pucB* promoter using a Hill function and in some complicated cases we derived a similar function by following Uri Alon et al., (2006) (4). We assume the following five possibilities for PpsR-*puc* binding (Fig.3A).

***Case (i)***

In this case, we assume that *in vivo* only oxidised PpsR (PpsR_ox_) is able to bind the promoter of *pucB,* and it represses *pucB* transcription. Then,

$${Level of pucB mRNA = M}_{max}\left( \frac{{K_{1}}^{n_{1}}}{{K_{1}}^{n_{1}}+{\mathrm{PpsR}_{\mathrm{ox}}}^{n_{1}}} \right) \left( 2 \right)$$

where M_max_ is maximum expression level of *pucB*, n_1_ is Hill coefficient and K_1_ is the EC_50_ value for binding between PpsR_ox_ and the *puc* promoter. The EC_50_ for this binding has been estimated to be 31 nM through *in vitro* experiments (2). From available microarray data we have obtained that the maximal mean relative *pucB* expression level under anaerobic conditions is 5.1 in arbitrary unit (a.u.). Therefore, we assign M_max_ = 5.1. We obtained n_1_ = 4.1 from the curve fitting of the data obtained from the isothermal curves published elsewhere (2) (Fig.3B).

Since one of the parameters of the model is blue light irradiance (dimensionless quantity *I*), to compare the model result with experimental gene expression pattern we assume a very low value of the blue light irradiance, *I=0.001.* In the existing simple model (1), for this light irradiance, blue light is not capable of repressing PS gene expression under semi-aerobic conditions. We find a parameter combination for which the simple model result is consistent with the micro-array data within the biologically meaningful range of oxygen (Fig. 4A).

Note that to obtain Fig. 4A, we have assumed that the total concentration of PpsR is 100 nM. Subsequently, we changed the total concentration of PpsR protein and obtained the changes in the shape of the transcriptomic profile (Fig. 4C). From comparisons of these profiles with the transcriptomic pattern obtained from the microarray data analysis we suggest that the total concentration of PpsR protein in a cell should be approximately 100 nM.

***Case (ii)***

In this case we assume that only PpsR_red_ binds the *pucB* promoter *in vivo* and acts as a repressor.

$${Level of pucB mRNA = M}_{\max}\left( \frac{{K_{2}}^{n_{2}}}{{K_{2}}^{n_{2}}+{\mathrm{PpsR}_{\mathrm{red}}}^{n_{2}}} \right) \left( 3 \right)$$

where n_2_ is the Hill coefficient and K_2_ is the EC_50_ for the binding of PpsR_red_ and DNA. An estimate of n_2_ = 3.4 is available from a previous study (3) and Masuda et. al (2) have estimated K_2_ = 69 nM. The parameter M_max_ represents maximal expression of *pucB* as in case (i). We tried several parameter combinations and found that it is not possible to get a transcriptomic pattern which is consistent with the micro-array data (Fig. 5 of the main text). In fact, we get a PS gene expression patterns in which PS genes are expressed even at high oxygen levels which is not consistent with the biological data.

***Case (iii)***

Here, we assume that only PpsR_red_ binds the *pucB* promoter and activates transcription.

$${Level of pucB mRNA = M}_{max}\left( \frac{{\mathrm{PpsR}_{\mathrm{red}}}^{n_{2}}}{{K_{2}}^{n_{2}}+{\mathrm{PpsR}_{\mathrm{red}}}^{n_{2}}} \right) \left( 4 \right)$$

where parameters M_max_, n_2_ and K_2_ are defined as in case (i) and case (ii). As shown in Fig. 6 of the main text, it is not possible to get a combination of parameters for which the model result is consistent with the microarray data. This suggests that activation of transcription by PpsR_red_ would not be sufficient to attain maximum PS gene expression in the absence of oxygen.

***Case (iv)***

For this case we assume that both PpsR_ox_ and PpsR_red_ are able to bind to the upstream region of *pucB* and both repress *pucB* expression. In what follows, we have derived an expression for the mRNA level of *pucB* for this possibility.

From the law of conservation

$O_{T}=$ *O + [*${OP}_{OX}$*] + [*${OP}_{red}$*]* (1)

where $O_{T}$= total concentration of PpsR binding sites on the *puc* promoter. The concentration of free binding sites is denoted by *O*. *[*${OP}_{OX}$*]* and *[*${OP}_{red}$*]* denote the concentration of binding sites bound to the oxidised and reduced forms of PpsR, respectively. Since we assume that the oxidised and reduced forms of PpsR bind to the same binding sites, the following binding events are possible.

*O + PpsR_ox_* $\begin{matrix} \underset{\to}{k_{1}^{+}} \\ \overset{\leftarrow}{k_{1}^{-}} \end{matrix}$ *[OP_ox_  ]* (2)

*O +*${PpsR}_{red}$ $\begin{matrix} \underset{\to}{k_{2}^{+}} \\ \overset{\leftarrow}{k_{2}^{-}} \end{matrix}$ *[OP_red_ ]* (3)

In chemical equilibrium,

$K_{1}=\frac{k_{1}^{-}}{k_{1}^{+}}$ =$\frac{O. {PpsR}_{ox}}{[OP_{ox}]}$ (4)

$K_{2}=\frac{k_{2}^{-}}{k_{2}^{+}}$ =$\frac{O. {PpsR}_{red}}{[OP_{red}]}$ (5)

Then, the fraction of free binding sites (*O/O_T_*) = $\frac{1}{1+\frac{{PpsR}_{ox}}{K_{1}}+\frac{{PpsR}_{red}}{K_{2}}}$(6)

Therefore, we write Hill function for this case as

*Level of pucB mRNA = M_max_* $\left( \frac{1}{1+\frac{{PpsR}_{ox}^{n_{1}}}{K_{1}^{n_{1}}}+\frac{{PpsR}_{red}^{n_{2}}}{K_{2}^{n_{2}}}} \right)$

In this case our model results are consistent with the microarray data for certain combinations of parameters (Fig. S2). However, these combinations are very similar to the simpler model (1).

***Case (v)***

Finally, we assume that both PpsR_ox_ and PpsR_red_ could bind upstream of *pucB* *in vivo*. But, PpsR_ox_ represses whereas PpsR_red_ activates *pucB* expression. Again, we derive an expression for the mRNA level transcribed from *pucB* as following

*O + PpsR_ox_* $\begin{matrix} \underset{\to}{k_{1}^{+}} \\ \overset{\leftarrow}{k_{1}^{-}} \end{matrix}$ *[OP_ox_  ]* (7)

*O +PpsR_red_* $\begin{matrix} \underset{\to}{k_{2}^{+}} \\ \overset{\leftarrow}{k_{2}^{-}} \end{matrix}$ *[OP_red_ ]* (8)

where O, *[OP_ox_  ]* and *[OP_red_ ]* are defined as in case (iv) and follow the same conservation relations (Eq. 1 of case (iv)).

In case of chemical equilibrium,

$K_{1}=\frac{k_{1}^{-}}{k_{1}^{+}}$ =$\frac{O. {PpsR}_{ox}}{[OP_{ox}]}$ (9)

$K_{2}=\frac{k_{2}^{-}}{k_{2}^{+}}$ =$\frac{O. {PpsR}_{red}}{[OP_{red}]}$ (10)

Fraction of binding sites either free or bound to activator (*O/O_T_ + [OP_red_]/O_T_* )

= $\frac{1+\frac{{PpsR}_{red}}{K_{2}}}{1+\frac{{PpsR}_{ox}}{K_{1}}+\frac{{PpsR}_{red}}{K_{2}}} ($11)

Therefore, we write the Hill function for this case as

*Level of pucB mRNA = M_max_* $\left( \frac{1+\frac{{PpsR}_{red}^{n_{2}}}{K_{2}^{n_{2}}}}{1+\frac{{PpsR}_{ox}^{n_{1}}}{K_{1}^{n_{1}}}+\frac{{PpsR}_{red}^{n_{2}}}{K_{2}^{n_{2}}}} \right)$

Fig. S3 shows that this mechanism is possible too, as there are parameters for which the gene expression profile from the simulations is consistent with the microarray data.

Overall, we have shown that PpsR_ox_ binding to the *pucB* promoter as a repressor is a minimal requirement for the models to fit the experimental data. Therefore, we continued to work with case (i), as this is the simplest model which fits the available experimental data significantly well.

**4 Figure S2**

**Figure S2. Case (iv), when both PpsR_ox_ and PpsR_red_ are able to bind the *puc* promoter and both act as repressor**. Similar to Fig. 5, 6 & 7 here we show how our model results vary if we change the model parameters by at least two orders of magnitudes from their default values. In panel A we vary *I*, in B the parameter α, in C the parameter β and in D the parameter γ. These results suggest that this possibility could lead to situations when the model results are consistent with the experimental data. Here, we calculate the normalised SSE for each simulation results in order to show the goodness of the fit. The default parameters are: *I* = 0.001, α = 6, β = 500, γ = 2.1, δ = 1, and K_eq_ =$\infty$. Here, we assume that the total concentration of PpsR is 100 nM as well as 30% O_2_ ≅ 200–380 μM and 0 % O_2_ = 0 μM.

**5 Figure S3**

**Figure S3. Case (v), when *in vivo* both oxidised and reduced PpsR are able to bind the promoter region of *pucB* but PpsR_ox_ represses whereas PpsR_red_ activates transcription.** Similar to Fig. 5, 6, 7 and S2 here we show how our model results vary if we increase or decrease the values of model parameters by at least two orders of magnitudes. In panel **a** we show effect of changes in *I*, in **b** effect of changes in α, in **c** effect of changes in β and effect of changes in γ are shown in **d**. Like Fig. S2 these results suggest that this possibility could also explain the experimental *pucB* gene expression pattern. The total concentration of PpsR is assumed 100 nM. The default parameters are: *I* = 0.001, α = 6, β = 500, γ = 2.1, δ = 1, and K_eq_ = $\infty$. In addition, we assume that 30% O_2_ ≅ 200–380 μM and 0 % O_2_ = 0 μM.

**References**

1. Pandey R, Flockerzi D, Hauser MJB, Straube R. Modeling the Light- and Redox-Dependent Interaction of PpsR/AppA in *Rhodobacter sphaeroides*. Biophys J. 2011;100:2347–55.

2. Masuda S, Bauer CE. AppA is a blue light photoreceptor that antirepresses photosynthesis gene expression in *Rhodobacter sphaeroides*. Cell. 2002;110:613–23.

3. Pandey R, Flockerzi D, Hauser MJB, Straube R. An extended model for the repression of photosynthesis genes by the AppA/PpsR system in *Rhodobacter sphaeroides*. FEBS J. 2012;279:3449–61.

4. Alon U. An introduction to systems biology: design principles of biological circuits. CRC press; 2006.
