# Supplementary material for: Use of transcriptomic data for extending a model of the AppA/PpsR system in Rhodobacter sphaeroides
Source: BMC Syst Biol. 2017 Dec 28;11:146. doi: 10.1186/s12918-017-0489-y (PMC5747161; doi:10.1186/s12918-017-0489-y)
Supplement: Supplementary file 2 — List of micro-array data series used for this study and data. (DOCX 118 kb) [file 12918_2017_489_MOESM2_ESM.docx]

|  |  |  | The complete data with data series (for GEO database) which have been used in the study. | | | | | | | |  |  |  |
| --- | --- | --- | --- | --- | --- | --- | --- | --- | --- | --- | --- | --- | --- |
|  |  |  |  |  |  |  |  |  |  |  |  |  |  |
|  |  |  |  |  |  |  |  |  |  |  |  |  |  |
|  |  | Note : There are four copies of *pucB* gene (RSP0314) with probe id 1194, 1195, 1196, 1197, 1198 | | | | | |  |  |  |  |  |  |
|  |  |  |  |  |  |  |  |  |  |  |  |  |  |
|  | **MAS5.0/RMA** | **Series** | **Probe Id** | **Data sample** | **Title** | **Environmental Condition** | **Expression Value** | **rpoZ** | **Relative Value** | **Mean** | **stdev** |  |  |
|  | MAS5.0 | published (Roh,2004), not submitted | 1194 | NA | 3W-Signal | anoxic, 3 W/M2 | 22297.76667 | 7073.733333 | 3.152192147 | 3.199579666 | 0.182635124 |  |  |
|  | MAS5.0 | published (Roh,2004), not submitted | 1195 | NA | 3W-Signal | anoxic, 3 W/M2 | 21950.4 | 7073.733333 | 3.103085594 |  |  |  |  |
|  | MAS5.0 | published (Roh,2004), not submitted | 1196 | NA | 3W-Signal | anoxic, 3 W/M2 | 24360.96667 | 7073.733333 | 3.443862741 |  |  |  |  |
|  | MAS5.0 | published (Roh,2004), not submitted | 1197 | NA | 3W-Signal | anoxic, 3 W/M2 | 21082.1 | 7073.733333 | 2.980335702 |  |  |  |  |
|  | MAS5.0 | published (Roh,2004), not submitted | 1198 | NA | 3W-Signal | anoxic, 3 W/M2 | 23473.63333 | 7073.733333 | 3.318422144 |  |  |  |  |
|  |  |  |  |  |  |  |  |  |  |  |  |  |  |
|  | MAS5.0 | GSE2145,GSE2150 | 1194 | GSM38773 | 2.4.1-1 | photo, 10 w/m-2 | 43443.3 | 12907 | 3.365871233 | 5.135059403 | 2.414470178 |  |  |
|  | MAS5.0 | GSE2145,GSE2150 | 1195 | GSM38773 | 2.4.1-1 | photo, 10 w/m-2 | 38088.4 | 12907 | 2.950987836 |  |  |  |  |
|  | MAS5.0 | GSE2145,GSE2150 | 1196 | GSM38773 | 2.4.1-1 | photo, 10 w/m-2 | 39657.3 | 12907 | 3.072542031 |  |  |  |  |
|  | MAS5.0 | GSE2145,GSE2150 | 1197 | GSM38773 | 2.4.1-1 | photo, 10 w/m-2 | 40547.6 | 12907 | 3.141520105 |  |  |  |  |
|  | MAS5.0 | GSE2145,GSE2150 | 1198 | GSM38773 | 2.4.1-1 | photo, 10 w/m-2 | 43282.3 | 12907 | 3.353397381 |  |  |  |  |
|  | MAS5.0 | GSE2145,GSE2150 | 1194 | GSM38774 | 2.4.1-3 | photo, 10 w/m-2 | 50427.7 | 12037.1 | 4.18935624 |  |  |  |  |
|  | MAS5.0 | GSE2145,GSE2150 | 1195 | GSM38774 | 2.4.1-3 | photo, 10 w/m-2 | 50500.6 | 12037.1 | 4.195412516 |  |  |  |  |
|  | MAS5.0 | GSE2145,GSE2150 | 1196 | GSM38774 | 2.4.1-3 | photo, 10 w/m-2 | 54397.4 | 12037.1 | 4.519144977 |  |  |  |  |
|  | MAS5.0 | GSE2145,GSE2150 | 1197 | GSM38774 | 2.4.1-3 | photo, 10 w/m-2 | 46948.2 | 12037.1 | 3.900291598 |  |  |  |  |
|  | MAS5.0 | GSE2145,GSE2150 | 1198 | GSM38774 | 2.4.1-3 | photo, 10 w/m-2 | 50756.5 | 12037.1 | 4.21667179 |  |  |  |  |
|  | MAS5.0 | GSE2145,GSE2150 | 1194 | GSM38775 | P3 | photo, 10 w/m-2 | 44754.6 | 12038.5 | 3.717622627 |  |  |  |  |
|  | MAS5.0 | GSE2145,GSE2150 | 1195 | GSM38775 | P3 | photo, 10 w/m-2 | 46694.6 | 12038.5 | 3.878772272 |  |  |  |  |
|  | MAS5.0 | GSE2145,GSE2150 | 1196 | GSM38775 | P3 | photo, 10 w/m-2 | 48643.5 | 12038.5 | 4.040661212 |  |  |  |  |
|  | MAS5.0 | GSE2145,GSE2150 | 1197 | GSM38775 | P3 | photo, 10 w/m-2 | 43382.1 | 12038.5 | 3.603613407 |  |  |  |  |
|  | MAS5.0 | GSE2145,GSE2150 | 1198 | GSM38775 | P3 | photo, 10 w/m-2 | 46906.9 | 12038.5 | 3.89640736 |  |  |  |  |
|  | MAS5.0 | GSE2145,GSE2150 | 1194 | GSM38776 | P5-2 | photo, 10 w/m-2 | 46542.8 | 10170.9 | 4.57607488 |  |  |  |  |
|  | MAS5.0 | GSE2145,GSE2150 | 1195 | GSM38776 | P5-2 | photo, 10 w/m-2 | 48390 | 10170.9 | 4.75769106 |  |  |  |  |
|  | MAS5.0 | GSE2145,GSE2150 | 1196 | GSM38776 | P5-2 | photo, 10 w/m-2 | 52313.6 | 10170.9 | 5.143458298 |  |  |  |  |
|  | MAS5.0 | GSE2145,GSE2150 | 1197 | GSM38776 | P5-2 | photo, 10 w/m-2 | 47107.8 | 10170.9 | 4.63162552 |  |  |  |  |
|  | MAS5.0 | GSE2145,GSE2150 | 1198 | GSM38776 | P5-2 | photo, 10 w/m-2 | 49226 | 10170.9 | 4.839886342 |  |  |  |  |
|  | MAS5.0 is written | GSE532,GSE8082 | 1194 | GSM2416 | 10--1 | anoxic photosynthetic and 10 W/m2 without salt stress | 22613.9 | 7028.4 | 3.217503272 |  |  |  |  |
|  | MAS5.0 is written | GSE532,GSE8082 | 1195 | GSM2416 | 10--1 | anoxic photosynthetic and 10 W/m2 without salt stress | 22843.5 | 7028.4 | 3.250170736 |  |  |  |  |
|  | MAS5.0 is written | GSE532,GSE8082 | 1196 | GSM2416 | 10--1 | anoxic photosynthetic and 10 W/m2 without salt stress | 23018.1 | 7028.4 | 3.275012805 |  |  |  |  |
|  | MAS5.0 is written | GSE532,GSE8082 | 1197 | GSM2416 | 10--1 | anoxic photosynthetic and 10 W/m2 without salt stress | 21176.8 | 7028.4 | 3.013032838 |  |  |  |  |
|  | MAS5.0 is written | GSE532,GSE8082 | 1198 | GSM2416 | 10---1 | anoxic photosynthetic and 10 W/m2 without salt stress | 20567.8 | 7028.4 | 2.926384383 |  |  |  |  |
|  | MAS5.0 is written | GSE532,GSE8082 | 1194 | GSM2417 | 10--2 | anoxic photosynthetic and 10 W/m2 without salt stress | 45411.4 | 7360.2 | 6.169859515 |  |  |  |  |
|  | MAS5.0 is written | GSE532,GSE8082 | 1195 | GSM2417 | 10--2 | anoxic photosynthetic and 10 W/m2 without salt stress | 44552.9 | 7360.2 | 6.053218663 |  |  |  |  |
|  | MAS5.0 is written | GSE532,GSE8082 | 1196 | GSM2417 | 10--2 | anoxic photosynthetic and 10 W/m2 without salt stress | 43602.4 | 7360.2 | 5.92407815 |  |  |  |  |
|  | MAS5.0 is written | GSE532,GSE8082 | 1197 | GSM2417 | 10--2 | anoxic photosynthetic and 10 W/m2 without salt stress | 42363 | 7360.2 | 5.755685987 |  |  |  |  |
|  | MAS5.0 is written | GSE532,GSE8082 | 1198 | GSM2417 | 10--2 | (10W/m2 white light illumination, no oxygen, no salt stress | 38966.7 | 7360.2 | 5.294244722 |  |  |  |  |
|  | MAS5.0 is written | GSE532,GSE8082 | 1194 | GSM8107 | 10--3 | no oxygen photosynthetic, 10 W/m2, without salt stress | 18065.4 | 5125.5 | 3.524612233 |  |  |  |  |
|  | MAS5.0 is written | GSE532,GSE8082 | 1195 | GSM8107 | 10--3 | no oxygen photosynthetic, 10 W/m2, without salt stress | 19710.9 | 5125.5 | 3.845654083 |  |  |  |  |
|  | MAS5.0 is written | GSE532,GSE8082 | 1196 | GSM8107 | 10--3 | no oxygen photosynthetic, 10 W/m2, without salt stress | 17641.7 | 5125.5 | 3.441947127 |  |  |  |  |
|  | MAS5.0 is written | GSE532,GSE8082 | 1197 | GSM8107 | 10--3 | no oxygen photosynthetic, 10 W/m2, without salt stress | 17680.4 | 5125.5 | 3.44949761 |  |  |  |  |
|  | MAS5.0 is written | GSE532,GSE8082 | 1198 | GSM8107 | 10-3, | no oxygen photosynthetic, 10 W/m2, without salt stress | 16091.5 | 5125.5 | 3.139498586 |  |  |  |  |
|  | MAS5.0 is written | GSE8082 | 1194 | GSM200619 | 10-4, | 10W/m2 white light illumination, no oxygen | 36493.5 | 3753.7 | 9.722007619 |  |  |  |  |
|  | MAS5.0 is written | GSE8082 | 1195 | GSM200619 | 10-4, | 10W/m2 white light illumination, no oxygen | 32476.2 | 3753.7 | 8.651783574 |  |  |  |  |
|  | MAS5.0 is written | GSE8082 | 1196 | GSM200619 | 10-4, | 10W/m2 white light illumination, no oxygen | 31825.3 | 3753.7 | 8.47838133 |  |  |  |  |
|  | MAS5.0 is written | GSE8082 | 1197 | GSM200619 | 10-4, | 10W/m2 white light illumination, no oxygen | 31388.9 | 3753.7 | 8.362122706 |  |  |  |  |
|  | MAS5.0 is written | GSE8082 | 1198 | GSM200619 | 10-4, | 10W/m2 white light illumination, no oxygen | 31963.5 | 3753.7 | 8.515198338 |  |  |  |  |
|  | MAS5.0 is written | GSE8082 | 1194 | GSM548557 | 2010_401 | at anoxic , 10 W/m2 illumination | 95068.2 | 10355 | 9.180898117 |  |  |  |  |
|  | MAS5.0 is written | GSE8082 | 1195 | GSM548557 | 2010_401 | at anoxic , 10 W/m2 illumination | 70349.6 | 10355 | 6.793780782 |  |  |  |  |
|  | MAS5.0 is written | GSE8082 | 1196 | GSM548557 | 2010_401 | at anoxic , 10 W/m2 illumination | 74935.1 | 10355 | 7.236610333 |  |  |  |  |
|  | MAS5.0 is written | GSE8082 | 1197 | GSM548557 | 2010_401 | at anoxic , 10 W/m2 illumination | 69770.1 | 10355 | 6.737817479 |  |  |  |  |
|  | MAS5.0 is written | GSE8082 | 1198 | GSM548557 | 2010_401 | at anoxic , 10 W/m2 illumination | 68886 | 10355 | 6.652438436 |  |  |  |  |
|  | Most Likely MAS5.0 | GSE532 | 1194 | GSM2427 | H-P1 | photosynthetic conditions ,10w/m-2 | 46745.4 | 6344 | 7.368442623 |  |  |  |  |
|  | Most Likely MAS5.0 | GSE532 | 1195 | GSM2427 | H-P1 | photosynthetic conditions,10w/m-2 | 56779 | 6344 | 8.950031526 |  |  |  |  |
|  | Most Likely MAS5.0 | GSE532 | 1196 | GSM2427 | H-P1 | photosynthetic conditions, ,10w/m-2 | 52490.6 | 6344 | 8.274054224 |  |  |  |  |
|  | Most Likely MAS5.0 | GSE532 | 1197 | GSM2427 | H-P1 | photosynthetic conditions, ,10w/m-2 | 47046.7 | 6344 | 7.415936318 |  |  |  |  |
|  | Most Likely MAS5.0 | GSE532 | 1198 | GSM2427 | H-P1 | photosynthetic conditions,10w/m-2 | 44038.6 | 6344 | 6.941771753 |  |  |  |  |
|  | Most Likely MAS5.0 | GSE532 | 1194 | GSM2428 | H-P2 | photosynthetic conditions,10w/m-2 | 43463.5 | 4284.9 | 10.14341058 |  |  |  |  |
|  | Most Likely MAS5.0 | GSE532 | 1195 | GSM2428 | H-P2 | photosynthetic conditions,,10w/m-2 | 46678.4 | 4284.9 | 10.89369647 |  |  |  |  |
|  | Most Likely MAS5.0 | GSE532 | 1196 | GSM2428 | H-P2 | photosynthetic conditions,10w/m-2 | 45642.6 | 4284.9 | 10.65196387 |  |  |  |  |
|  | Most Likely MAS5.0 | GSE532 | 1197 | GSM2428 | H-P2 | photosynthetic conditions,10w/m-2 | 43672 | 4284.9 | 10.19206983 |  |  |  |  |
|  | Most Likely MAS5.0 | GSE532 | 1198 | GSM2428 | H-P2 | photosynthetic conditions,10w/m-2 | 6264.9 | 4284.9 | 1.462087797 |  |  |  |  |
| Absolute Call criteria is same | Most Likely MAS5.0 | GSE139 | 1194 | GSM3258 | 10W_1_19Dc02 | anaerobic, 10 W/M2 | 17751.3 | 6928.3 | 2.562143672 |  |  |  |  |
| Absolute Call criteria is same | Most Likely MAS5.0 | GSE139 | 1195 | GSM3258 | 10W_1_19Dc02 | anaerobic, 10 W/M2 | 16423.4 | 6928.3 | 2.370480493 |  |  |  |  |
| Absolute Call criteria is same | Most Likely MAS5.0 | GSE139 | 1196 | GSM3258 | 10W_1_19Dc02 | anaerobic, 10 W/M2 | 19933.5 | 6928.3 | 2.877112712 |  |  |  |  |
| Absolute Call criteria is same | Most Likely MAS5.0 | GSE139 | 1197 | GSM3258 | 10W_1_19Dc02 | anaerobic, 10 W/M2 | 16237.1 | 6928.3 | 2.34359078 |  |  |  |  |
| Absolute Call criteria is same | Most Likely MAS5.0 | GSE139 | 1198 | GSM3258 | 10W_1_19Dc02 | anaerobic, 10 W/M2 | 19019 | 6928.3 | 2.74511785 |  |  |  |  |
| Absolute Call criteria is same | Most Likely MAS5.0 | GSE139 | 1194 | GSM3260 | 10W_2_19Dc02 | anaerobic, 10 W/M2 | 21697.7 | 7138.7 | 3.039446958 |  |  |  |  |
| Absolute Call criteria is same | Most Likely MAS5.0 | GSE139 | 1195 | GSM3260 | 10W_2_19Dc02 | anaerobic, 10 W/M2 | 22229 | 7138.7 | 3.113872274 |  |  |  |  |
| Absolute Call criteria is same | Most Likely MAS5.0 | GSE139 | 1196 | GSM3260 | 10W_2_19Dc02 | anaerobic, 10 W/M2 | 24764.7 | 7138.7 | 3.469077003 |  |  |  |  |
| Absolute Call criteria is same | Most Likely MAS5.0 | GSE139 | 1197 | GSM3260 | 10W_2_19Dc02 | anaerobic, 10 W/M2 | 21019.2 | 7138.7 | 2.944401642 |  |  |  |  |
| Absolute Call criteria is same | Most Likely MAS5.0 | GSE139 | 1198 | GSM3260 | 10W_2_19Dc02 | anaerobic, 10 W/M2 | 25156.5 | 7138.7 | 3.523960945 |  |  |  |  |
| Absolute Call criteria is same | Most Likely MAS5.0 | GSE139 | 1194 | GSM3262 | 10W_3_19Dc02 | anaerobic, 10 W/M2 | 22680.1 | 7988.6 | 2.839058158 |  |  |  |  |
| Absolute Call criteria is same | Most Likely MAS5.0 | GSE139 | 1195 | GSM3262 | 10W_3_19Dc02 | anaerobic, 10 W/M2 | 21260.7 | 7988.6 | 2.661379966 |  |  |  |  |
| Absolute Call criteria is same | Most Likely MAS5.0 | GSE139 | 1196 | GSM3262 | 10W_3_19Dc02 | anaerobic, 10 W/M2 | 25926.5 | 7988.6 | 3.245437248 |  |  |  |  |
| Absolute Call criteria is same | Most Likely MAS5.0 | GSE139 | 1197 | GSM3262 | 10W_3_19Dc02 | anaerobic, 10 W/M2 | 21000.3 | 7988.6 | 2.628783517 |  |  |  |  |
| Absolute Call criteria is same | Most Likely MAS5.0 | GSE139 | 1198 | GSM3262 | 10W_3_19Dc02 | anaerobic, 10 W/M2 | 26231.6 | 7988.6 | 3.283629172 |  |  |  |  |
|  | MAS5.0 is written | GSE12269 | 1194 | GSM308084 | before shift, biological rep1 | anaerobic, 10 W/M2 | 16889 | 2172 | 7.775782689 |  |  |  |  |
|  | MAS5.0 is written | GSE12269 | 1195 | GSM308084 | before shift, biological rep1 | anaerobic, 10 W/M2 | 16164.4 | 2172 | 7.442173112 |  |  |  |  |
|  | MAS5.0 is written | GSE12269 | 1196 | GSM308084 | before shift, biological rep1 | anaerobic, 10 W/M2 | 16539.1 | 2172 | 7.614686924 |  |  |  |  |
|  | MAS5.0 is written | GSE12269 | 1197 | GSM308084 | before shift, biological rep1 | anaerobic, 10 W/M2 | 14786.3 | 2172 | 6.807688766 |  |  |  |  |
|  | MAS5.0 is written | GSE12269 | 1198 | GSM308084 | before shift, biological rep1 | anaerobic, 10 W/M2 | 15086.1 | 2172 | 6.945718232 |  |  |  |  |
|  |  |  |  |  |  |  |  |  |  |  |  |  |  |
|  | MAS5.0 | published (Roh,2004), not submitted | 1194 | NA | 100W-Signal | anoxic, 100 W/M2 | 12351.46667 | 5333.1 | 2.316001325 | 2.248830886 | 0.156968368 |  |  |
|  | MAS5.0 | published (Roh,2004), not submitted | 1195 | NA | 100W-Signal | anoxic, 100 W/M2 | 12061.66667 | 5333.1 | 2.261661448 |  |  |  |  |
|  | MAS5.0 | published (Roh,2004), not submitted | 1196 | NA | 100W-Signal | anoxic, 100 W/M2 | 12695.66667 | 5333.1 | 2.380541649 |  |  |  |  |
|  | MAS5.0 | published (Roh,2004), not submitted | 1197 | NA | 100W-Signal | anoxic, 100 W/M2 | 10551.4 | 5333.1 | 1.978474058 |  |  |  |  |
|  | MAS5.0 | published (Roh,2004), not submitted | 1198 | NA | 100W-Signal | anoxic, 100 W/M2 | 12306 | 5333.1 | 2.307475952 |  |  |  |  |
|  |  |  |  |  |  |  |  |  |  |  |  |  |  |
| Absolute Call criteria is same | MAS 5.0 SINCE GSE7004 is sure | GSE1515,GSE7004 | 1194 | GSM27348 | 0.5A | 0.5% O2 in dark | 25024.3 | 5801.2 | 4.313642005 | 3.210155627 | 0.749447992 |  |  |
| Absolute Call criteria is same | MAS 5.0 SINCE GSE7004 is sure | GSE1515,GSE7004 | 1195 | GSM27348 | 0.5A | 0.5% O2 in dark | 26004.2 | 5801.2 | 4.482555333 |  |  |  |  |
| Absolute Call criteria is same | MAS 5.0 SINCE GSE7004 is sure | GSE1515,GSE7004 | 1196 | GSM27348 | 0.5A | 0.5% O2 in dark | 24818.8 | 5801.2 | 4.2782183 |  |  |  |  |
| Absolute Call criteria is same | MAS 5.0 SINCE GSE7004 is sure | GSE1515,GSE7004 | 1197 | GSM27348 | 0.5A | 0.5% O2 in dark | 24620.6 | 5801.2 | 4.244052955 |  |  |  |  |
| Absolute Call criteria is same | MAS 5.0 SINCE GSE7004 is sure | GSE1515,GSE7004 | 1198 | GSM27348 | 0.5A | 0.5% O2 in dark | 21278 | 5801.2 | 3.667861822 |  |  |  |  |
| Absolute Call criteria is same | MAS 5.0 SINCE GSE7004 is sure | GSE1515,GSE7004 | 1194 | GSM27349 | 0.5C | 0.5% O2 in dark | 23075.3 | 8715.3 | 2.647677074 |  |  |  |  |
| Absolute Call criteria is same | MAS 5.0 SINCE GSE7004 is sure | GSE1515,GSE7004 | 1195 | GSM27349 | 0.5C | 0.5% O2 in dark | 24299.7 | 8715.3 | 2.78816564 |  |  |  |  |
| Absolute Call criteria is same | MAS 5.0 SINCE GSE7004 is sure | GSE1515,GSE7004 | 1196 | GSM27349 | 0.5C | 0.5% O2 in dark | 24121 | 8715.3 | 2.767661469 |  |  |  |  |
| Absolute Call criteria is same | MAS 5.0 SINCE GSE7004 is sure | GSE1515,GSE7004 | 1197 | GSM27349 | 0.5C | 0.5% O2 in dark | 22479.9 | 8715.3 | 2.579360435 |  |  |  |  |
| Absolute Call criteria is same | MAS 5.0 SINCE GSE7004 is sure | GSE1515,GSE7004 | 1198 | GSM27349 | 0.5C | 0.5% O2 in dark | 21496.4 | 8715.3 | 2.466512914 |  |  |  |  |
| Absolute Call criteria is same | MAS 5.0 SINCE GSE7004 is sure | GSE1515,GSE7004 | 1194 | GSM27350 | 0.5D | 0.5% O2 in dark | 21404.8 | 7460.4 | 2.869122299 |  |  |  |  |
| Absolute Call criteria is same | MAS 5.0 SINCE GSE7004 is sure | GSE1515,GSE7004 | 1195 | GSM27350 | 0.5D | 0.5% O2 in dark | 21404.8 | 7460.4 | 2.869122299 |  |  |  |  |
| Absolute Call criteria is same | MAS 5.0 SINCE GSE7004 is sure | GSE1515,GSE7004 | 1196 | GSM27350 | 0.5D | 0.5% O2 in dark | 21399.6 | 7460.4 | 2.868425286 |  |  |  |  |
| Absolute Call criteria is same | MAS 5.0 SINCE GSE7004 is sure | GSE1515,GSE7004 | 1197 | GSM27350 | 0.5D | 0.5% O2 in dark | 19685.1 | 7460.4 | 2.638611871 |  |  |  |  |
| Absolute Call criteria is same | MAS 5.0 SINCE GSE7004 is sure | GSE1515,GSE7004 | 1198 | GSM27350 | 0.5D | 0.5% O2 in dark | 19929.3 | 7460.4 | 2.6713447 |  |  |  |  |
|  |  |  |  |  |  |  |  |  |  |  |  |  |  |
|  | MAS 5.0 | GSE33641 | 1194 | GSM832034 | R. sphaeroides 2.4.1, untreated rep1 | 2% O2 | 19382 | 3552.1 | 5.456490527 | 6.965080429 | 1.635206725 |  |  |
|  | MAS 5.0 | GSE33641 | 1195 | GSM832034 | R. sphaeroides 2.4.1, untreated rep1 | 2% O2 | 19612.6 | 3552.1 | 5.52140987 |  |  |  |  |
|  | MAS 5.0 | GSE33641 | 1196 | GSM832034 | R. sphaeroides 2.4.1, untreated rep1 | 2% O2 | 19943.1 | 3552.1 | 5.614453422 |  |  |  |  |
|  | MAS 5.0 | GSE33641 | 1197 | GSM832034 | R. sphaeroides 2.4.1, untreated rep1 | 2% O2 | 18790.1 | 3552.1 | 5.289856704 |  |  |  |  |
|  | MAS 5.0 | GSE33641 | 1198 | GSM832034 | R. sphaeroides 2.4.1, untreated rep1 | 2% O2 | 18565.9 | 3552.1 | 5.226739112 |  |  |  |  |
|  | MAS 5.0 | GSE33641 | 1194 | GSM832035 | R. sphaeroides 2.4.1, untreated rep2 | 2% O2 | 29615.6 | 3479.9 | 8.510474439 |  |  |  |  |
|  | MAS 5.0 | GSE33641 | 1195 | GSM832035 | R. sphaeroides 2.4.1, untreated rep2 | 2% O2 | 29407.3 | 3479.9 | 8.450616397 |  |  |  |  |
|  | MAS 5.0 | GSE33641 | 1196 | GSM832035 | R. sphaeroides 2.4.1, untreated rep2 | 2% O2 | 30734.2 | 3479.9 | 8.831920457 |  |  |  |  |
|  | MAS 5.0 | GSE33641 | 1197 | GSM832035 | R. sphaeroides 2.4.1, untreated rep2 | 2% O2 | 29158.8 | 3479.9 | 8.379206299 |  |  |  |  |
|  | MAS 5.0 | GSE33641 | 1198 | GSM832035 | R. sphaeroides 2.4.1, untreated rep2 | 2% O2 | 29125.5 | 3479.9 | 8.369637059 |  |  |  |  |
|  |  |  |  |  |  |  |  |  |  |  |  |  |  |
|  | MAS 5.0 | GSE2150,GSE2146 | 1194 | GSM38780 | 2.4.1-A1 | 3%oxygen, dark | 37309.5 | 9801 | 3.806703398 | 3.566581023 | 1.511546106 |  |  |
|  | MAS 5.0 | GSE2150,GSE2146 | 1195 | GSM38780 | 2.4.1-A1 | 3%oxygen, dark | 35161.2 | 9801 | 3.587511478 |  |  |  |  |
|  | MAS 5.0 | GSE2150,GSE2146 | 1196 | GSM38780 | 2.4.1-A1 | 3%oxygen, dark | 38274.1 | 9801 | 3.905121926 |  |  |  |  |
|  | MAS 5.0 | GSE2150,GSE2146 | 1197 | GSM38780 | 2.4.1-A1 | 3%oxygen, dark | 36900.4 | 9801 | 3.764962759 |  |  |  |  |
|  | MAS 5.0 | GSE2150,GSE2146 | 1198 | GSM38780 | 2.4.1-A1 | 3%oxygen, dark | 37487.1 | 9801 | 3.824823998 |  |  |  |  |
|  | MAS 5.0 | GSE2150,GSE2146 | 1194 | GSM38781 | 2.4.1-B2 | 3%oxygen, dark | 28294.4 | 10081.4 | 2.806594322 |  |  |  |  |
|  | MAS 5.0 | GSE2150,GSE2146 | 1195 | GSM38781 | 2.4.1-B2 | 3%oxygen, dark | 27726.6 | 10081.4 | 2.750272780 |  |  |  |  |
|  | MAS 5.0 | GSE2150,GSE2146 | 1196 | GSM38781 | 2.4.1-B2 | 3%oxygen, dark | 27794.3 | 10081.4 | 2.756988117 |  |  |  |  |
|  | MAS 5.0 | GSE2150,GSE2146 | 1197 | GSM38781 | 2.4.1-B2 | 3%oxygen, dark | 25705.2 | 10081.4 | 2.549764914 |  |  |  |  |
|  | MAS 5.0 | GSE2150,GSE2146 | 1198 | GSM38781 | 2.4.1-B2 | 3%oxygen, dark | 27043.4 | 10081.4 | 2.682504414 |  |  |  |  |
|  | MAS 5.0 SINCE GSE7004 is sure | GSE532,GSE7004 | 1194 | GSM1672 | S2 | 3 % O2 , dark | 14200.5 | 5001.9 | 2.839021172 |  |  |  |  |
|  | MAS 5.0 SINCE GSE7004 is sure | GSE532,GSE7004 | 1195 | GSM1672 | S2 | 3 % O2 , dark | 14083.5 | 5001.9 | 2.815630061 |  |  |  |  |
|  | MAS 5.0 SINCE GSE7004 is sure | GSE532,GSE7004 | 1196 | GSM1672 | S2 | 3 % O2 , dark | 14293.6 | 5001.9 | 2.857634099 |  |  |  |  |
|  | MAS 5.0 SINCE GSE7004 is sure | GSE532,GSE7004 | 1197 | GSM1672 | S2 | 3 % O2 , dark | 12837.2 | 5001.9 | 2.566464743 |  |  |  |  |
|  | MAS 5.0 SINCE GSE7004 is sure | GSE532,GSE7004 | 1198 | GSM1672 | S2 | 3 % O2 , dark | 12476.5 | 5001.9 | 2.494352146 |  |  |  |  |
|  | MAS 5.0 SINCE GSE7004 is sure | GSE532,GSE7004 | 1194 | GSM1673 | S3 | 3 % O2, dark | 15624.6 | 5727.8 | 2.727853626 |  |  |  |  |
|  | MAS 5.0 SINCE GSE7004 is sure | GSE532,GSE7004 | 1195 | GSM1673 | S3 | 3 % O2, dark | 16252.2 | 5727.8 | 2.837424491 |  |  |  |  |
|  | MAS 5.0 SINCE GSE7004 is sure | GSE532,GSE7004 | 1196 | GSM1673 | S3 | 3 % O2, dark | 15946.3 | 5727.8 | 2.784018297 |  |  |  |  |
|  | MAS 5.0 SINCE GSE7004 is sure | GSE532,GSE7004 | 1197 | GSM1673 | S3 | 3 % O2, dark | 15109.3 | 5727.8 | 2.637888893 |  |  |  |  |
|  | MAS 5.0 SINCE GSE7004 is sure | GSE532,GSE7004 | 1198 | GSM1673 | S3 | 3 % O2, dark | 14094.5 | 5727.8 | 2.460717902 |  |  |  |  |
|  | Most Likely MAS5.0 | GSE532 | 1194 | GSM2425 | H-S2 | semiaerobic conditions,3% O2 | 28198.2 | 4789.3 | 5.887749776 |  |  |  |  |
|  | Most Likely MAS5.0 | GSE532 | 1195 | GSM2425 | H-S2 | semiaerobic conditions,3% O2 | 34386.8 | 4789.3 | 7.179921909 |  |  |  |  |
|  | Most Likely MAS5.0 | GSE532 | 1196 | GSM2425 | H-S2 | semiaerobic conditions,3% O2 | 32973.8 | 4789.3 | 6.884889232 |  |  |  |  |
|  | Most Likely MAS5.0 | GSE532 | 1197 | GSM2425 | H-S2 | semiaerobic conditions,3% O2 | 25901.7 | 4789.3 | 5.408243376 |  |  |  |  |
|  | Most Likely MAS5.0 | GSE532 | 1198 | GSM2425 | H-S2 | semiaerobic conditions,3% O2 | 27419.4 | 4789.3 | 5.725137285 |  |  |  |  |
|  | Most Likely MAS5.0 | GSE532 | 1194 | GSM2426 | H-S4 | semiaerobic conditions,3% O2 | 22610.4 | 4393.5 | 5.146329805 |  |  |  |  |
|  | Most Likely MAS5.0 | GSE532 | 1195 | GSM2426 | H-S4 | semiaerobic conditions,3% O2 | 21787.1 | 4393.5 | 4.958939342 |  |  |  |  |
|  | Most Likely MAS5.0 | GSE532 | 1196 | GSM2426 | H-S4 | semiaerobic conditions,3% O2 | 22587.5 | 4393.5 | 5.141117560 |  |  |  |  |
|  | Most Likely MAS5.0 | GSE532 | 1197 | GSM2426 | H-S4 | semiaerobic conditions,3% O2 | 23003.4 | 4393.5 | 5.235780130 |  |  |  |  |
|  | Most Likely MAS5.0 | GSE532 | 1198 | GSM2426 | H-S4 | semiaerobic conditions,3% O2 | 22255.7 | 4393.5 | 5.065596905 |  |  |  |  |
|  | MAS5.0 | GSE532 | 1194 | GSM161565 | S4 | semiaerobically, 3% Oxygen | 10453.2 | 5783 | 1.807573924 |  |  |  |  |
|  | MAS5.0 | GSE532 | 1195 | GSM161565 | S4 | semiaerobically, 3% Oxygen | 10536.3 | 5783 | 1.821943628 |  |  |  |  |
|  | MAS5.0 | GSE532 | 1196 | GSM161565 | S4 | semiaerobically, 3% Oxygen | 10033.8 | 5783 | 1.735051012 |  |  |  |  |
|  | MAS5.0 | GSE532 | 1197 | GSM161565 | S4 | semiaerobically, 3% Oxygen | 10068.1 | 5783 | 1.740982189 |  |  |  |  |
|  | MAS5.0 | GSE532 | 1198 | GSM161565 | S4 | semiaerobically, 3% Oxygen | 9454.2 | 5783 | 1.634826215 |  |  |  |  |
|  |  |  |  |  |  |  |  |  |  |  |  |  |  |
|  | Most Likely MAS5.0 | GSE1480,GSE7004 | 1194 | GSM25295 | 10A | 10% O2, dark | 6114.1 | 7466.9 | 0.81882709 | 1.200207071 | 0.421114185 |  |  |
|  | Most Likely MAS5.0 | GSE1480,GSE7004 | 1195 | GSM25295 | 10A | 10% O2, dark | 5867.2 | 7466.9 | 0.785761159 |  |  |  |  |
|  | Most Likely MAS5.0 | GSE1480,GSE7004 | 1196 | GSM25295 | 10A | 10% O2, dark | 6425.5 | 7466.9 | 0.860531144 |  |  |  |  |
|  | Most Likely MAS5.0 | GSE1480,GSE7004 | 1197 | GSM25295 | 10A | 10% O2, dark | 5705.4 | 7466.9 | 0.764092194 |  |  |  |  |
|  | Most Likely MAS5.0 | GSE1480,GSE7004 | 1198 | GSM25295 | 10A | 10% O2, dark | 5611.9 | 7466.9 | 0.751570263 |  |  |  |  |
|  | MAS5.0 | GSE1480,GSE7004 | 1194 | GSM25296 | 10B | 10% O2, dark | 5866.2 | 5173.5 | 1.133893882 |  |  |  |  |
|  | MAS5.0 | GSE1480,GSE7004 | 1195 | GSM25296 | 10B | 10% O2, dark | 5696.9 | 5173.5 | 1.101169421 |  |  |  |  |
|  | MAS5.0 | GSE1480,GSE7004 | 1196 | GSM25296 | 10B | 10% O2, dark | 5190.4 | 5173.5 | 1.003266647 |  |  |  |  |
|  | MAS5.0 | GSE1480,GSE7004 | 1197 | GSM25296 | 10B | 10% O2, dark | 5379.5 | 5173.5 | 1.039818305 |  |  |  |  |
|  | MAS5.0 | GSE1480,GSE7004 | 1198 | GSM25296 | 10B | 10% O2, dark | 5213.8 | 5173.5 | 1.007789697 |  |  |  |  |
|  | MAS5.0 | GSE1480,GSE7004 | 1194 | GSM25297 | 10BL-dark | 10% O2, dark | 11582.8 | 6107.8 | 1.896394774 |  |  |  |  |
|  | MAS5.0 | GSE1480,GSE7004 | 1195 | GSM25297 | 10BL-dark | 10% O2, dark | 10518.4 | 6107.8 | 1.722125806 |  |  |  |  |
|  | MAS5.0 | GSE1480,GSE7004 | 1196 | GSM25297 | 10BL-dark | 10% O2, dark | 11097.8 | 6107.8 | 1.816988114 |  |  |  |  |
|  | MAS5.0 | GSE1480,GSE7004 | 1197 | GSM25297 | 10BL-dark | 10% O2, dark | 10078.5 | 6107.8 | 1.650103147 |  |  |  |  |
|  | MAS5.0 | GSE1480,GSE7004 | 1198 | GSM25297 | 10BL-dark | 10% O2, dark | 10082.6 | 6107.8 | 1.65077442 |  |  |  |  |
|  |  |  |  |  |  |  |  |  |  |  |  |  |  |
|  | MAS5.0 | GSE1480 | 1194 | GSM25302 | BL-135A | 10% O2, bluelight | 1521.3 | 5927.3 | 0.256659862 | 0.215877335 | 0.03728485 |  |  |
|  | MAS5.0 | GSE1480 | 1195 | GSM25302 | BL-135A | 10% O2, bluelight | 1539.2 | 5927.3 | 0.259679787 |  |  |  |  |
|  | MAS5.0 | GSE1480 | 1196 | GSM25302 | BL-135A | 10% O2, bluelight | 1425 | 5927.3 | 0.240413004 |  |  |  |  |
|  | MAS5.0 | GSE1480 | 1197 | GSM25302 | BL-135A | 10% O2, bluelight | 1422.6 | 5927.3 | 0.240008098 |  |  |  |  |
|  | MAS5.0 | GSE1480 | 1198 | GSM25302 | BL-135A | 10% O2, bluelight | 1308.1 | 5927.3 | 0.220690702 |  |  |  |  |
|  | MAS5.0 | GSE1480 | 1194 | GSM25303 | BL-135B | 10% O2, bluelight | 2063.2 | 8534.7 | 0.241742533 |  |  |  |  |
|  | MAS5.0 | GSE1480 | 1195 | GSM25303 | BL-135B | 10% O2, bluelight | 1481.5 | 8534.7 | 0.17358548 |  |  |  |  |
|  | MAS5.0 | GSE1480 | 1196 | GSM25303 | BL-135B | 10% O2, bluelight | 1597.7 | 8534.7 | 0.187200487 |  |  |  |  |
|  | MAS5.0 | GSE1480 | 1197 | GSM25303 | BL-135B | 10% O2, bluelight | 1512.2 | 8534.7 | 0.177182561 |  |  |  |  |
|  | MAS5.0 | GSE1480 | 1198 | GSM25303 | BL-135B | 10% O2, bluelight | 1379.3 | 8534.7 | 0.161610836 |  |  |  |  |
|  |  |  |  |  |  |  |  |  |  |  |  |  |  |
|  | Most Likely MAS5.0 since GSE7004 is | GSE1515,GSE7004 | 1194 | GSM26242 | 20A | 20% O2 and dark | 1365.4 | 6241.7 | 0.218754506 | 0.209038633 | 0.092603929 |  |  |
|  | Most Likely MAS5.0 since GSE7004 is | GSE1515,GSE7004 | 1195 | GSM26242 | 20A | 20% O2 and dark | 1218.8 | 6241.7 | 0.195267315 |  |  |  |  |
|  | Most Likely MAS5.0 since GSE7004 is | GSE1515,GSE7004 | 1196 | GSM26242 | 20A | 20% O2 and dark | 1318.5 | 6241.7 | 0.211240527 |  |  |  |  |
|  | Most Likely MAS5.0 since GSE7004 is | GSE1515,GSE7004 | 1197 | GSM26242 | 20A | 20% O2 and dark | 1066.6 | 6241.7 | 0.170882933 |  |  |  |  |
|  | Most Likely MAS5.0 since GSE7004 is | GSE1515,GSE7004 | 1198 | GSM26242 | 20A | 20% O2 and dark | 1132.7 | 6241.7 | 0.181472996 |  |  |  |  |
|  | Most Likely MAS5.0 since GSE7004 is | GSE1515,GSE7004 | 1194 | GSM26243 | 20B | 20% O2 and dark | 2914.1 | 8009.3 | 0.363839537 |  |  |  |  |
|  | Most Likely MAS5.0 since GSE7004 is | GSE1515,GSE7004 | 1195 | GSM26243 | 20B | 20% O2 and dark | 2675.6 | 8009.3 | 0.334061653 |  |  |  |  |
|  | Most Likely MAS5.0 since GSE7004 is | GSE1515,GSE7004 | 1196 | GSM26243 | 20B | 20% O2 and dark | 2362.5 | 8009.3 | 0.294969598 |  |  |  |  |
|  | Most Likely MAS5.0 since GSE7004 is | GSE1515,GSE7004 | 1197 | GSM26243 | 20B | 20% O2 and dark | 2603.8 | 8009.3 | 0.325097075 |  |  |  |  |
|  | Most Likely MAS5.0 since GSE7004 is | GSE1515,GSE7004 | 1198 | GSM26243 | 20B | 20% O2 and dark | 2341 | 8009.3 | 0.292285218 |  |  |  |  |
|  | Most Likely MAS5.0 since GSE7004 is | GSE1515,GSE7004 | 1194 | GSM26244 | 20C | 20% O2 and dark | 912 | 7269.2 | 0.125460849 |  |  |  |  |
|  | Most Likely MAS5.0 since GSE7004 is | GSE1515,GSE7004 | 1195 | GSM26244 | 20C | 20% O2 and dark | 778.3 | 7269.2 | 0.107068178 |  |  |  |  |
|  | Most Likely MAS5.0 since GSE7004 is | GSE1515,GSE7004 | 1196 | GSM26244 | 20C | 20% O2 and dark | 875.1 | 7269.2 | 0.120384637 |  |  |  |  |
|  | Most Likely MAS5.0 since GSE7004 is | GSE1515,GSE7004 | 1197 | GSM26244 | 20C | 20% O2 and dark | 697.6 | 7269.2 | 0.095966544 |  |  |  |  |
|  | Most Likely MAS5.0 since GSE7004 is | GSE1515,GSE7004 | 1198 | GSM26244 | 20C | 20% O2 and dark | 718.4 | 7269.2 | 0.098827932 |  |  |  |  |
|  |  |  |  |  |  |  |  |  |  |  |  |  |  |
|  |  |  |  |  |  |  |  |  |  |  |  |  |  |
|  | Most Likely MAS5.0 since GSE7004 is | GSE532,GSE7004 | 1194 | GSM1670 | G_1 | 30 % O2, dark | 803 | 6290.9 | 0.127644693 | 0.239427459 | 0.172485714 |  |  |
|  | Most Likely MAS5.0 since GSE7004 is | GSE532,GSE7004 | 1195 | GSM1670 | G_1 | 30 % O2, dark | 645.7 | 6290.9 | 0.102640322 |  |  |  |  |
|  | Most Likely MAS5.0 since GSE7004 is | GSE532,GSE7004 | 1196 | GSM1670 | G_1 | 30 % O2, dark | 728.1 | 6290.9 | 0.115738607 |  |  |  |  |
|  | Most Likely MAS5.0 since GSE7004 is | GSE532,GSE7004 | 1197 | GSM1670 | G_1 | 30 % O2, dark | 761 | 6290.9 | 0.120968383 |  |  |  |  |
|  | Most Likely MAS5.0 since GSE7004 is | GSE532,GSE7004 | 1198 | GSM1670 | G_1 | 30 % O2, dark | 564.5 | 6290.9 | 0.089732789 |  |  |  |  |
|  | Most Likely MAS5.0 since GSE7004 is | GSE532,GSE7004 | 1194 | GSM1671 | G_5 | 30 % O2 dark | 1010 | 6049.7 | 0.166950427 |  |  |  |  |
|  | Most Likely MAS5.0 since GSE7004 is | GSE532,GSE7004 | 1195 | GSM1671 | G_5 | 30 % O2 dark | 853.6 | 6049.7 | 0.141097906 |  |  |  |  |
|  | Most Likely MAS5.0 since GSE7004 is | GSE532,GSE7004 | 1196 | GSM1671 | G_5 | 30 % O2 dark | 926.2 | 6049.7 | 0.153098501 |  |  |  |  |
|  | Most Likely MAS5.0 since GSE7004 is | GSE532,GSE7004 | 1197 | GSM1671 | G_5 | 30 % O2 dark | 684.5 | 6049.7 | 0.113146106 |  |  |  |  |
|  | Most Likely MAS5.0 since GSE7004 is | GSE532,GSE7004 | 1198 | GSM1671 | G_5 | 30 % O2 dark | 818.4 | 6049.7 | 0.081657903 |  |  |  |  |
|  | Most Likely MAS5.0 since GSE7004 is | GSE532,GSE7004 | 1194 | GSM8108 | 30C | 30 % O2 dark | 427.7 | 10022.3 | 0.042674835 |  |  |  |  |
|  | Most Likely MAS5.0 since GSE7004 is | GSE532,GSE7004 | 1195 | GSM8108 | 30C | 30 % O2 dark | 418.4 | 10022.3 | 0.041746904 |  |  |  |  |
|  | Most Likely MAS5.0 since GSE7004 is | GSE532,GSE7004 | 1196 | GSM8108 | 30C | 30 % O2 dark | 408.6 | 10022.3 | 0.040769085 |  |  |  |  |
|  | Most Likely MAS5.0 since GSE7004 is | GSE532,GSE7004 | 1197 | GSM8108 | 30C | 30 % O2 dark | 311.4 | 10022.3 | 0.031070712 |  |  |  |  |
|  | Most Likely MAS5.0 since GSE7004 is | GSE532,GSE7004 | 1198 | GSM8108 | 30C | 30 % O2 dark | 356.8 | 10022.3 | 0.035600611 |  |  |  |  |
|  | Most Likely MAS5.0 | GSE532 | 1194 | GSM2420 | H-A1 | aerobic conditions, 30 % O2 | 3991.1 | 6090.8 | 0.65526696 |  |  |  |  |
|  | Most Likely MAS5.0 | GSE532 | 1195 | GSM2420 | H-A1 | aerobic conditions, 30 % O2 | 3156.9 | 6090.8 | 0.518306298 |  |  |  |  |
|  | Most Likely MAS5.0 | GSE532 | 1196 | GSM2420 | H-A1 | aerobic conditions, 30 % O2 | 3536.9 | 6090.8 | 0.580695475 |  |  |  |  |
|  | Most Likely MAS5.0 | GSE532 | 1197 | GSM2420 | H-A1 | aerobic conditions, 30 % O2 | 2574.1 | 6090.8 | 0.422621002 |  |  |  |  |
|  | Most Likely MAS5.0 | GSE532 | 1198 | GSM2420 | H-A1 | aerobic conditions, 30 % O2 | 3010.2 | 6090.8 | 0.494220792 |  |  |  |  |
|  | Most Likely MAS5.0 | GSE532 | 1194 | GSM2421 | H-A4 | aerobic conditions, 30 % O2 | 4454.4 | 8360.5 | 0.532791101 |  |  |  |  |
|  | Most Likely MAS5.0 | GSE532 | 1195 | GSM2421 | H-A4 | aerobic conditions, 30 % O2 | 4328.3 | 8360.5 | 0.517708271 |  |  |  |  |
|  | Most Likely MAS5.0 | GSE532 | 1196 | GSM2421 | H-A4 | aerobic conditions, 30 % O2 | 4517.6 | 8360.5 | 0.540350458 |  |  |  |  |
|  | Most Likely MAS5.0 | GSE532 | 1197 | GSM2421 | H-A4 | aerobic conditions, 30 % O2 | 3865.7 | 8360.5 | 0.462376652 |  |  |  |  |
|  | Most Likely MAS5.0 | GSE532 | 1198 | GSM2421 | H-A4 | aerobic conditions, 30 % O2 | 3831.7 | 8360.5 | 0.45830991 |  |  |  |  |
|  | Most Likely MAS5.0 | GSE532 | 1194 | GSM2422 | H-1R | aerobic conditions, 30 % O2 | 1224.9 | 7675.6 | 0.159583616 |  |  |  |  |
|  | Most Likely MAS5.0 | GSE532 | 1195 | GSM2422 | H-1R | aerobic conditions, 30 % O2 | 1411.8 | 7675.6 | 0.183933504 |  |  |  |  |
|  | Most Likely MAS5.0 | GSE532 | 1196 | GSM2422 | H-1R | aerobic conditions, 30 % O2 | 1342.5 | 7675.6 | 0.174904893 |  |  |  |  |
|  | Most Likely MAS5.0 | GSE532 | 1197 | GSM2422 | H-1R | aerobic conditions, 30 % O2 | 1081.5 | 7675.6 | 0.140901037 |  |  |  |  |
|  | Most Likely MAS5.0 | GSE532 | 1198 | GSM2422 | H-1R | aerobic conditions , 30 % O2 | 939.9 | 7675.6 | 0.138430269 |  |  |  |  |
|  | Most Likely MAS5.0 | GSE532 | 1194 | GSM2423 | H-2R | aerobic conditions, 30 % O2 | 3740.3 | 6789.7 | 0.550878537 |  |  |  |  |
|  | Most Likely MAS5.0 | GSE532 | 1195 | GSM2423 | H-2R | aerobic conditions, 30 % O2 | 3709.5 | 6789.7 | 0.546342254 |  |  |  |  |
|  | Most Likely MAS5.0 | GSE532 | 1196 | GSM2423 | H-2R | aerobic conditions, 30 % O2 | 3465.2 | 6789.7 | 0.510361283 |  |  |  |  |
|  | Most Likely MAS5.0 | GSE532 | 1197 | GSM2423 | H-2R | aerobic conditions, 30 % O2 | 3028.8 | 6789.7 | 0.446087456 |  |  |  |  |
|  | Most Likely MAS5.0 | GSE532 | 1198 | GSM2423 | H-2R | aerobic conditions, 30 % O2 | 2764.7 | 6789.7 | 0.407190303 |  |  |  |  |
|  | MAS5.0 | GSE2145, GSE2150, GSE2219 | 1194 | GSM38777 | wt1 | 30 %oxygen,dark | 2382.5 | 9635.1 | 0.247272991 |  |  |  |  |
|  | MAS5.0 | GSE2145, GSE2150, GSE2219 | 1195 | GSM38777 | wt1 | 30 %oxygen,dark | 2072.8 | 9635.1 | 0.215130097 |  |  |  |  |
|  | MAS5.0 | GSE2145, GSE2150, GSE2219 | 1196 | GSM38777 | wt1 | 30 %oxygen,dark | 2168.7 | 9635.1 | 0.225083289 |  |  |  |  |
|  | MAS5.0 | GSE2145, GSE2150, GSE2219 | 1197 | GSM38777 | wt1 | 30 %oxygen,dark | 1822.2 | 9635.1 | 0.189121026 |  |  |  |  |
|  | MAS5.0 | GSE2145, GSE2150, GSE2219 | 1198 | GSM38777 | wt1 | 30 %oxygen,dark | 1936.1 | 9635.1 | 0.200942388 |  |  |  |  |
|  | MAS5.0 | GSE2145,GSE2150 | 1194 | GSM38778 | 2.4.1 A | 30 %oxygen,dark | 1650.4 | 9720.4 | 0.169787252 |  |  |  |  |
|  | MAS5.0 | GSE2145,GSE2150 | 1195 | GSM38778 | 2.4.1 A | 30 %oxygen,dark | 1749.6 | 9720.4 | 0.179992593 |  |  |  |  |
|  | MAS5.0 | GSE2145,GSE2150 | 1196 | GSM38778 | 2.4.1 A | 30 %oxygen,dark | 1723.9 | 9720.4 | 0.177348669 |  |  |  |  |
|  | MAS5.0 | GSE2145,GSE2150 | 1197 | GSM38778 | 2.4.1 A | 30 %oxygen,dark | 876.6 | 9720.4 | 0.090181474 |  |  |  |  |
|  | MAS5.0 | GSE2145,GSE2150 | 1198 | GSM38778 | 2.4.1 A | 30 %oxygen,dark | 1777.6 | 9720.4 | 0.182873133 |  |  |  |  |
|  | MAS5.0 | GSE2145,GSE2150 | 1194 | GSM38779 | 2.4.1 B | 30 %oxygen,dark | 2090.1 | 10564.1 | 0.19784932 |  |  |  |  |
|  | MAS5.0 | GSE2145,GSE2150 | 1195 | GSM38779 | 2.4.1 B | 30 %oxygen,dark | 1964.7 | 10564.1 | 0.185978929 |  |  |  |  |
|  | MAS5.0 | GSE2145,GSE2150 | 1196 | GSM38779 | 2.4.1 B | 30 %oxygen,dark | 2435.5 | 10564.1 | 0.230544959 |  |  |  |  |
|  | MAS5.0 | GSE2145,GSE2150 | 1197 | GSM38779 | 2.4.1 B | 30 %oxygen,dark | 1986.6 | 10564.1 | 0.188051987 |  |  |  |  |
|  | MAS5.0 | GSE2145,GSE2150 | 1198 | GSM38779 | 2.4.1 B | 30 %oxygen,dark | 1817.1 | 10564.1 | 0.172007081 |  |  |  |  |
|  | MAS5.0 | published (Roh,2004), not submitted | 1194 | NA | H-A_31Oct02_Signal | 30 % oxygen | 1348.5 | 6119.933333 | 0.220345538 |  |  |  |  |
|  | MAS5.0 | published (Roh,2004), not submitted | 1195 | NA | H-A_31Oct02_Signal | 30 % oxygen | 1155.6 | 6119.933333 | 0.188825586 |  |  |  |  |
|  | MAS5.0 | published (Roh,2004), not submitted | 1196 | NA | H-A_31Oct02_Signal | 30 % oxygen | 1271.333333 | 6119.933333 | 0.207736468 |  |  |  |  |
|  | MAS5.0 | published (Roh,2004), not submitted | 1197 | NA | H-A_31Oct02_Signal | 30 % oxygen | 947.4666667 | 6119.933333 | 0.154816501 |  |  |  |  |
|  | MAS5.0 | published (Roh,2004), not submitted | 1198 | NA | H-A_31Oct02_Signal | 30 % oxygen | 1155.2 | 6119.933333 | 0.188760226 |  |  |  |  |
|  | MAS5.0 | GSE532 | 1194 | GSM3030 | Houston-Aerobic-1 | 30% oxygen | 474.7 | 5945.7 | 0.079839212 |  |  |  |  |
|  | MAS5.0 | GSE532 | 1195 | GSM3030 | Houston-Aerobic-1 | 30% oxygen | 406.1 | 5945.7 | 0.068301462 |  |  |  |  |
|  | MAS5.0 | GSE532 | 1196 | GSM3030 | Houston-Aerobic-1 | 30% oxygen | 458.6 | 5945.7 | 0.077131372 |  |  |  |  |
|  | MAS5.0 | GSE532 | 1197 | GSM3030 | Houston-Aerobic-1 | 30% oxygen | 367.6 | 5945.7 | 0.061826194 |  |  |  |  |
|  | MAS5.0 | GSE532 | 1198 | GSM3030 | Houston-Aerobic-1 | 30% oxygen | 459.3 | 5945.7 | 0.077249104 |  |  |  |  |
|  | MAS5.0 | GSE532 | 1194 | GSM3031 | Houston-Aerobic-3 | 30% oxygen | 888.6 | 7109.1 | 0.124994725 |  |  |  |  |
|  | MAS5.0 | GSE532 | 1195 | GSM3031 | Houston-Aerobic-3 | 30% oxygen | 673 | 7109.1 | 0.094667398 |  |  |  |  |
|  | MAS5.0 | GSE532 | 1196 | GSM3031 | Houston-Aerobic-3 | 30% oxygen | 827.6 | 7109.1 | 0.116414173 |  |  |  |  |
|  | MAS5.0 | GSE532 | 1197 | GSM3031 | Houston-Aerobic-3 | 30% oxygen | 618.9 | 7109.1 | 0.087057433 |  |  |  |  |
|  | MAS5.0 | GSE532 | 1198 | GSM3031 | Houston-Aerobic-3 | 30% oxygen | 725.7 | 7109.1 | 0.102080432 |  |  |  |  |
|  | MAS5.0 | GSE532 | 1194 | GSM3032 | Houston-Aerobic-4 | 30% oxygen | 2682.2 | 5305 | 0.505598492 |  |  |  |  |
|  | MAS5.0 | GSE532 | 1195 | GSM3032 | Houston-Aerobic-4 | 30% oxygen | 2387.7 | 5305 | 0.450084826 |  |  |  |  |
|  | MAS5.0 | GSE532 | 1196 | GSM3032 | Houston-Aerobic-4 | 30% oxygen | 2527.8 | 5305 | 0.476493874 |  |  |  |  |
|  | MAS5.0 | GSE532 | 1197 | GSM3032 | Houston-Aerobic-4 | 30% oxygen | 1855.9 | 5305 | 0.349839774 |  |  |  |  |
|  | MAS5.0 | GSE532 | 1198 | GSM3032 | Houston-Aerobic-4 | 30% oxygen | 2280.6 | 5305 | 0.429896324 |  |  |  |  |
|  |  |  |  |  |  |  |  |  |  |  |  |  |  |
|  |  |  |  |  |  |  |  |  |  |  |  |  |  |
